# Document retrieval on repetitive string collections

**DOI:** 10.1007/s10791-017-9297-7

**Published:** 2017-04-01

**Authors:** Travis Gagie, Aleksi Hartikainen, Kalle Karhu, Juha Kärkkäinen, Gonzalo Navarro, Simon J. Puglisi, Jouni Sirén

**Affiliations:** 1 CeBiB — Center of Biotechnology and Bioengineering, School of Computer Science and Telecommunications, Diego Portales University, Santiago, Chile; 2 Google Inc, Mountain View, CA USA; 3 Research and Technology, Planmeca Oy, Helsinki, Finland; 4 Department of Computer Science, Helsinki Institute of Information Technology, University of Helsinki, Helsinki, Finland; 5 Department of Computer Science, CeBiB — Center of Biotechnology and Bioengineering, University of Chile, Santiago, Chile; 6 Wellcome Trust Sanger Institute, Cambridge, UK

**Keywords:** Repetitive string collections, Document retrieval on strings, Suffix trees and arrays

## Abstract

Most of the fastest-growing string collections today are repetitive, that is, most of the constituent documents are similar to many others. As these collections keep growing, a key approach to handling them is to exploit their repetitiveness, which can reduce their space usage by orders of magnitude. We study the problem of indexing repetitive string collections in order to perform efficient document retrieval operations on them. Document retrieval problems are routinely solved by search engines on large natural language collections, but the techniques are less developed on generic string collections. The case of repetitive string collections is even less understood, and there are very few existing solutions. We develop two novel ideas, *interleaved LCPs* and *precomputed document lists*, that yield highly compressed indexes solving the problem of document listing (find all the documents where a string appears), top-*k* document retrieval (find the *k* documents where a string appears most often), and document counting (count the number of documents where a string appears). We also show that a classical data structure supporting the latter query becomes highly compressible on repetitive data. Finally, we show how the tools we developed can be combined to solve ranked conjunctive and disjunctive multi-term queries under the simple ${\textsf{tf}}{\textsf{-}}{\textsf{idf}}$ model of relevance. We thoroughly evaluate the resulting techniques in various real-life repetitiveness scenarios, and recommend the best choices for each case.

## Introduction

Document retrieval on natural language text collections is a routine activity in web and enterprise search engines. It is solved with variants of the inverted index (Büttcher et al. 2010; Baeza-Yates and Ribeiro-Neto 2011), an immensely successful technology that can by now be considered mature. The inverted index has well-known limitations, however: the text must be easy to parse into *terms* or *words*, and queries must be sets of words or sequences of words (*phrases*). Those limitations are acceptable in most cases when *natural language* text collections are indexed, and they enable the use of an extremely simple index organization that is efficient and scalable, and that has been the key to the success of Web-scale information retrieval.

Those limitations, on the other hand, hamper the use of the inverted index in other kinds of string collections where partitioning the text into words and limiting queries to word sequences is inconvenient, difficult, or meaningless: DNA and protein sequences, source code, music streams, and even some East Asian languages. Document retrieval queries are of interest in those string collections, but the state of the art about alternatives to the inverted index is much less developed (Hon et al. 2013; Navarro 2014).

In this article we focus on *repetitive string collections*, where most of the strings are very similar to many others. These types of collections arise naturally in scenarios like versioned document collections (such as Wikipedia^1^ or the Wayback Machine^2^), versioned software repositories, periodical data publications in text form (where very similar data is published over and over), sequence databases with genomes of individuals of the same species (which differ at relatively few positions), and so on. Such collections are the fastest-growing ones today. For example, genome sequencing data is expected to grow at least as fast as astronomical, YouTube, or Twitter data by 2025, exceeding Moore’s Law rate by a wide margin (Stephens et al. 2015). This growth brings new scientific opportunities but it also creates new computational problems.

A key tool for handling this kind of growth is to exploit repetitiveness to obtain size reductions of orders of magnitude. An appropriate Lempel-Ziv compressor^3^ can successfully capture such repetitiveness, and version control systems have offered direct access to any version since their beginnings, by means of storing the edits of a version with respect to some other version that is stored in full (Rochkind 1975). However, document retrieval requires much more than retrieving individual documents. In this article we focus on three basic document retrieval problems on string collections:

*Document Listing:* Given a string *P*, list the identifiers of all the ${\textsf{df}}$ documents where *P* appears.
*Top-k Retrieval:* Given a string *P* and *k*, list *k* documents where *P* appears most often.
*Document Counting:* Given a string *P*, return the number ${\textsf{df}}$ of documents where *P* appears.

Apart from the obvious case of information retrieval on East Asian and other languages where separating words is difficult, these queries are relevant in many other applications where string collections are maintained. For example, in pan-genomics (Marschall et al. 2016) we index the genomes of all the strains of an organism. The index can be either a specialized data structure, such as a colored de Bruijn graph, or a text index over the concatenation of the individual genomes. The parts of the genome common to all strains are called core; the parts common to several strains are called peripheral; and the parts in only one strain are called unique. Given a set of DNA reads from an unidentified strain, we may want to identify it (if it is known) or find the closest strain in our database (if it is not), by identifying reads from unique or peripheral genomes (i.e., those that occur rarely) and listing the corresponding strains. This boils down to document listing and counting problems. In turn, top-*k* retrieval is at the core of information retrieval systems, since the *term frequency* ${\textsf{tf}}$ (i.e., the number of times a pattern appears in a document) is a basic criterion to establish the relevance of a document for a query (Büttcher et al. 2010; Baeza-Yates and Ribeiro-Neto 2011). On multi-term queries, it is usually combined with the document frequency, ${\textsf{df}}$, to compute ${\textsf{tf}}{\textsf{-}}{\textsf{idf}}$, a simple and popular relevance model. Document counting is also important for data mining applications on strings (or *string mining* (Dhaliwal et al. 2012)), where the value ${\textsf{df}}/d$ of a given pattern, *d* being the total number of documents, is its *support* in the collection. Finally, we will show that the best choice of document listing and top-*k* retrieval algorithms in practice strongly depends on the ${\textsf{df}}/ occ$, where *occ* is the number of times the pattern appears in the collection, and thus the ability to compute ${\textsf{df}}$ quickly allows for the efficient selection of an appropriate listing or top-*k* algorithm at query time. Navarro (2014) lists several other applications of these queries.

In the case of natural language, there exist various proposals to reduce the inverted index size by exploiting the text repetitiveness (Anick and Flynn 1992; Broder et al. 2006; He et al. 2009, 2010; He and Suel 2012; Claude et al. 2016). For general string collections, the situation is much worse. Most of the indexing structures designed for repetitive string collections (Mäkinen et al. 2010; Claude et al. 2010; Claude and Navarro 2010, 2012; Kreft and Navarro 2013; Gagie et al. 2012a, 2014; Do et al. 2014; Belazzougui et al. 2015) support only *pattern matching*, that is, they count or list the *occ * occurrences of a pattern *P* in the whole collection. Of course one can retrieve the *occ * occurrences and then answer any of our three document retrieval queries, but the time will be *Ω*(*occ*). Instead, there are optimal-time indexes for string collections that solve document listing in time ${\mathcal {O}}\left( {|P |+{\textsf{df}}} \right)$ (Muthukrishnan 2002), top-*k* retrieval in time ${\mathcal {O}}\left( {|P |+k} \right)$ (Navarro and Nekrich 2012), and document counting in time ${\mathcal {O}}\left( {|P |} \right)$ (Sadakane 2007). The first two solutions, however, use a lot of space even for classical, non-repetitive collections. While more compact representations have been studied (Hon et al. 2013; Navarro 2014), none of those is tailored to the repetitive scenario, except for a grammar-based index that solves document listing (Claude and Munro 2013).

In this article we develop several novel solutions for the three document retrieval queries of interest, tailored to repetitive string collections. Our first idea, called *interleaved LCPs (ILCP)* stores the longest common prefix (LCP) array of the documents, interleaved in the order of the global LCP array. The ILCP turns out to have a number of interesting properties that make it compressible on repetitive collections, and useful for document listing and counting. Our second idea, *precomputed document lists (PDL)*, samples some nodes in the global suffix tree of the collection and stores precomputed answers on those. It then applies grammar compression on the stored answers, which is effective when the collection is repetitive. PDL yields very efficient solutions for document listing and top-*k* retrieval. Third, we show that a solution for document counting (Sadakane 2007) that uses just two bits per symbol (bps) in the worst case (which is unacceptably high in the repetitive scenario) turns out to be highly compressible when the collection is repetitive, and becomes the most attractive solution for document counting. Finally, we show how the different components of our solutions can be assembled to offer ${\textsf{tf}}{\textsf{-}}{\textsf{idf}}$ ranked conjunctive and disjunctive multi-term queries on repetitive string collections.

We implement and experimentally compare several variants of our solutions with the state of the art, including the solution for repetitive string collections (Claude and Munro 2013) and some relevant solutions for general string collections (Ferrada and Navarro 2013; Gog and Navarro 2015a). We consider various kinds of real-life repetitiveness scenarios, and show which solutions are the best depending on the kind and amount of repetitiveness, and the space reduction that can be achieved. For example, on very repetitive collections of up to 1 GB we perform document listing and top-*k* retrieval in 10–100 microseconds per result and using 1–2 bits per symbol. For counting, we use as little as 0.1 bits per symbol and answer queries in less than a microsecond. Multi-term top-*k* queries can be solved with a throughput of 100–200 queries per second, which we show to be similar to that of a state-of-the-art inverted index. Of course, we do not aim to compete with inverted indexes in the scenarios where they can be applied (mainly, in natural language text collections), but to offer similar functionality in the case of generic string collections, where inverted indexes cannot be used.

This article collects our earlier results appearing in *CPM 2013* (Gagie et al. 2013), *ESA 2014* (Navarro et al. 2014a), and *DCC 2015* (Gagie et al. 2015), where we focused on exploiting repetitiveness in different ways to handle different document retrieval problems. Here we present them in a unified form, considering the application of two new techniques (ILCP and PDL) and an existing one (Sadakane 2007) to the three problems (document listing, top-*k* retrieval, and document counting), and showing how they interact (e.g., the need to use fast document counting to choose the best document listing method). In this article we also consider a more complex document retrieval problem we had not addressed before: top-*k* retrieval of multi-word queries. We present an algorithm that uses our (single-term) top-*k* retrieval and document counting structures to solve ranked multi-term conjunctive and disjunctive queries under the ${\textsf{tf}}{\textsf{-}}{\textsf{idf}}$ relevance model.

The article is organized as follows (see Table 1). In Sect. 2 we introduce the concepts needed to follow the presentation. In Sect. 3 we introduce the Interleaved LCP (ILCP) structure and show how it can be used for document listing and, with a different representation, for document counting. In Sect. 4 we introduce our second structure, Precomputed Document Lists (PDL), and describe how it can be used for document listing and, with some reordering of the lists, for top-*k* retrieval. Section 5 then returns to the problem of document counting, not to propose a new data structure but to study a known one (Sadakane 2007), which is found to be compressible in a repetitiveness scenario (and, curiously, on totally random texts as well). Section 6 shows how our developments can be combined to build a document retrieval index that handles multi-term queries. Section 7 empirically studies the performance of our solutions on the three document retrieval problems, also comparing them with the state of the art for generic string collections, repetitive or not, and giving recommendations on which structure to use in each case. Finally, Sect. 8 concludes and gives some future work directions.

**Table 1:** The techniques we study and the document retrieval problems we solve with them

Problem	ILCP	PDL	Sadakane
Listing	Section 3.3	Section 4.1	
Top-*k*		Section 4.2	
Counting	Section 3.4		Section 5

## Preliminaries

### Suffix trees and arrays

A large number of solutions for pattern matching or document retrieval on string collections rely on the suffix tree (Weiner 1973) or the suffix array (Manber and Myers 1993). Assume that we have a collection of *d* strings, each terminated with a special symbol “$” (which we consider to be lexicographically smaller than any other symbol), and let *T*[1..*n*] be their concatenation. The suffix tree of *T* is a compacted digital tree where all the suffixes *T*[*i*..*n*] are inserted. Collecting the leaves of the suffix tree yields the suffix array, $${\textsf{SA}}[1..n]$$, which is an array of pointers to all the suffixes sorted in increasing lexicographic order, that is, $$T[{\textsf{SA}}[i]..n] < T[{\textsf{SA}}[i+1]..n]$$ for all 1 ≤ *i* < *n*. To find all the *occ* occurrences of a string *P*[1..*m*] in the collection, we traverse the suffix tree following the symbols of *P* and output the leaves of the node we arrive at, called the *locus* of *P*, in time $${\mathcal {O}}\left( {m+ occ } \right)$$. On a suffix array, we obtain the range $${\textsf{SA}}[\ell ..r]$$ of the leaves (i.e., of the suffixes prefixed by *P*) by binary search, and then list the contents of the range, in total time $${\mathcal {O}}\left( {m\lg n+ occ } \right)$$.

We will make use of compressed suffix arrays (Navarro and Mäkinen 2007), which we will call generically ${\textsf{CSA}}s$. Their size in bits is denoted $|{\textsf{CSA}} |$, their time to find ℓ and *r* is denoted ${\textsf{search}}\left( {m} \right)$, and their time to access any cell ${\textsf{SA}}[i]$ is denoted ${\textsf{lookup}}\left( {n} \right)$. A particular version of the ${\textsf{CSA}}$ that is tailored for repetitive collections is the Run-Length Compressed Suffix Array (RLCSA) (Mäkinen et al. 2010).

### Rank and select on sequences

Let *S*[1..*n*] be a sequence over an alphabet [1..*σ*]. When *σ* = 2 we use 0 and 1 as the two symbols, and the sequence is called a bitvector. Two operations of interest on *S* are ${{\textsf{rank}}}_c(S,i)$, which counts the number of occurrences of symbol *c* in *S*[1..*i*], and ${{\textsf{select}}}_c(S,j)$, which gives the position of the *j*th occurrence of symbol *c* in *S*. For bitvectors, one can compute both functions in ${\mathcal {O}}\left( {1} \right)$ time using *o*(*n*) bits on top of *S* (Clark 1996). If *S* contains *m* 1s, we can also represent it using $m\lg \frac{n}{m}+{\mathcal {O}}\left( {m} \right)$ bits, so that ${{\textsf{rank}}}$ takes ${\mathcal {O}}\left( {\lg \frac{n}{m}} \right)$ time and ${{\textsf{select}}}$ takes ${\mathcal {O}}\left( {1} \right)$ (Okanohara and Sadakane 2007).^4^

The wavelet tree (Grossi et al. 2003) is a tool for extending bitvector representations to sequences. It is a binary tree where the alphabet [1..*σ*] is recursively partitioned. The root represents *S* and stores a bitvector *W*[1..*n*] where *W*[*i*] = 0 iff symbol *S*[*i*] belongs to the left child. Left and right children represent a subsequence of *S* formed by the symbols of [1..*σ*] they handle, so they recursively store a bitvector and so on until reaching the leaves, which represent a single symbol. By giving constant-time ${{\textsf{rank}}}$ and ${{\textsf{select}}}$ capabilities to the bitvectors associated with the nodes, the wavelet tree can compute any *S*[*i*] = *c*, ${{\textsf{rank}}}_c(S,i)$, or ${{\textsf{select}}}_c(S,j)$) in time proportional to the depth of the leaf of *c*. If the bitvectors are represented in a certain compressed form (Raman et al. 2007), then the total space is at most $n\lg \sigma + o(nh)$, where *h* is the wavelet tree height, independent of the way the alphabet is partitioned (Grossi et al. 2003).

### Document listing

Let us now describe the optimal-time algorithm of Muthukrishnan (2002) for document listing. Muthukrishnan stores the suffix tree of *T*; a so-called *document array* ${\textsf{DA}}[1..n]$ of *T*, in which each cell ${\textsf{DA}}[i]$ stores the identifier of the document containing $T [{\textsf{SA}}[i]]$; an array *C*[1..*n*], in which each cell *C*[*i*] stores the largest value *h* < *i* such that ${\textsf{DA}}[h] = {\textsf{DA}}[i]$, or 0 if there is no such value *h*; and a data structure supporting range-minimum queries (RMQs) over *C*, ${\textsf{rmq}}_C(i,j) = \arg \min _{i \le k \le j} C[k]$. These data structures take a total of ${\mathcal {O}}\left( {n \lg n} \right)$ bits. Given a pattern *P*[1..*m*], the suffix tree is used to find the interval ${\textsf{SA}}[\ell..r]$ that contains the starting positions of the suffixes prefixed by *P*. It follows that every value *C*[*i*] < ℓ in *C*[ℓ..*r*] corresponds to a distinct document in ${\textsf{DA}}[i]$. Thus a recursive algorithm finding all those positions *i* starts with $k = {\textsf{rmq}}_C(\ell ,r)$. If $C[k] \ge \ell$ it stops. Otherwise it reports document ${\textsf{DA}}[k]$ and continues recursively with the ranges C[ℓ..*k* − 1] and *C*[*K*+1..*r*] (the condition $C[k] \ge \ell$ always uses the original ℓ value). In total, the algorithm uses ${\mathcal {O}}\left( {m + {\textsf{df}}} \right)$ time, where ${\textsf{df}}$ is the number of documents returned.

Sadakane (2007) proposed a space-efficient version of this algorithm, using just $|{\textsf{CSA}}|+{\mathcal {O}}\left( {n} \right)$ bits. The suffix tree is replaced with a ${\textsf{CSA}}$. The array ${\textsf{DA}}$ is replaced with a bitvector *B*[1..*n*] such that *B*[*i*] = 1 iff *i* is the first symbol of a document in *T*. Therefore ${\textsf{DA}}[i] = {{\textsf{rank}}}_1(B,{\textsf{SA}}[i])$ can be computed in constant time (Clark 1996). The RMQ data structure is replaced with a variant (Fischer and Heun 2011) that uses just 2*n* + *o*(*n*) bits and answers queries in constant time without accessing *C*. Finally, the comparisons $C[k] \ge \ell$ are replaced by marking the documents already reported in a bitvector *V*[1..*d*] (initially all 0s), so that $V[{\textsf{DA}}[i]]=1$ iff document ${\textsf{DA}}[i]$ has already been reported. If $V[{\textsf{DA}}[i]]=1$ the recursion stops, otherwise it sets $V[{\textsf{DA}}[i]]$, reports ${\textsf{DA}}[i]$, and continues. This is correct as long as the RMQ structure returns the leftmost minimum in the range, and the range [ℓ..*k* − 1] is processed before the range *C*[*K* + 1..*r*] (Navarro 2014). The total time is then ${\mathcal {O}}\left( {\textsf{search}\left( {m} \right) +{\textsf{df}}\cdot {\textsf{lookup}}\left( {n} \right) } \right)$.

## Interleaved LCP

We introduce our first structure, the Interleaved LCP (ILCP). The main idea is to interleave the longest-common-prefix (LCP) arrays of the documents, in the order given by the global LCP of the collection. This yields long runs of equal values on repetitive collections, making the ILCP structure run-length compressible. Then, we show that the classical document listing technique of Muthukrishnan (2002), designed to work on a completely different array, works almost verbatim over the ILCP array, and this yields a new document listing technique of independent interest for string collections. Finally, we show that a particular representation of the ILCP array allows us to count the number of documents where a string appears without having to list them one by one.

### The ILCP array

The longest-common-prefix array ${{\textsf{LCP}}}_S [1..|S |]$ of a string *S* is defined such that ${{\textsf{LCP}}}_S [1] = 0$ and, for $2 \le i \le |S |$, ${{\textsf{LCP}}}_S [i]$ is the length of the longest common prefix of the lexicographically (*i* − 1)th and *i*th suffixes of *S*, that is, of $S[{\textsf{SA}}_S[i-1]..|S |]$ and $S[{\textsf{SA}}_S[i]..|S |]$, where ${\textsf{SA}}_S$ is the suffix array of *S*. We define the interleaved LCP array of *T*, ${{\textsf{ILCP}}}$, to be the interleaving of the LCP arrays of the individual documents according to the document array.

#### **Definition 1**

Let $T[1..n] = S_1 \cdot S_2 \cdots S_d$ be the concatenation of documents *S*_*j*_, ${\textsf{DA}}$ the document array of *T*, and ${{\textsf{LCP}}}_{S_j}$ the longest-common-prefix array of string *S*_*j*_. Then the *interleaved LCP array of T* is defined, for all $1 \le i \le n$, as

$${{\textsf{ILCP}}}[i] = {{\textsf{LCP}}}_{S_{{\textsf{DA}}[i]}}\left[ {{\textsf{rank}}}_{{\textsf{DA}}[i]}({\textsf{DA}},i) \right] .$$

That is, if the suffix ${\textsf{SA}}[i]$ belongs to document *S*_*j*_ (i.e., ${\textsf{DA}}[i]=j$), and this is the *r*th suffix of ${\textsf{SA}}$ that belongs to *S*_*j*_ (i.e., $r = {\textsf{rank}}_j({\textsf{DA}},i)$), then ${{\textsf{ILCP}}}[i] = {{\textsf{LCP}}}_{S_j}[r]$. Therefore the order of the individual ${\textsf{LCP}}$ arrays is preserved in ${\textsf{ILCP}}$.

*Example* Consider the documents $S_1={\texttt {''TATA\$''}}$, $S_2={\texttt {''LATA\$''}}$, and $S_3={\texttt {''AAAA\$''}}$. Their concatenation is $T={\texttt {''TATA\$LATA\$AAAA\$''}}$, its suffix array is ${\textsf{SA}}=\langle 15,10,5,14,9,4,13,12,11,7,2,6,8,3,1\rangle$ and its document array is ${\textsf{DA}}=\langle 3 ,{{\textsf{2}}},1, 3 ,{{\textsf{2}}},1, 3 , 3 , 3 ,{{\textsf{2}}},1,{{\textsf{2}}},{{\textsf{2}}},1,1\rangle$. The LCP arrays of the documents are ${\textsf{LCP}}_{S_1}=\langle 0,0,1,0,2\rangle$, ${\textsf{LCP}}_{S_2}=\langle {{\textsf{0}}},{{\textsf{0}}},{{\textsf{1}}},{{\textsf{0}}},{{\textsf{0}}}\rangle$, and ${\textsf{LCP}}_{S_3}=\langle 0 , 0 , 1 , 2 , 3 \rangle$. Therefore, ${\textsf{ILCP}}= \langle 0 ,{\textsf{0}},0, 0 ,{\textsf{0}},0, 1 , 2 , 3 ,{\textsf{1}},1,{\textsf{0}},{\textsf{0}},0,2\rangle$ interleaves the ${\textsf{LCP}}$ arrays in the order given by ${\textsf{DA}}$ (notice the fonts above).

The following property of ${\textsf{ILCP}}$ makes it suitable for document retrieval.

#### **Lemma 1**

*Let* $T[1..n] = S_1 \cdot S_2 \cdots S_d$ *be the concatenation of documents* *S*_*j*_, ${\textsf{SA}}$ *its suffix array and* ${\textsf{DA}}$ *its document array. Let* ${\textsf{SA}}[\ell ..r]$ *be the interval that contains the starting positions of suffixes prefixed by a pattern* $P[1..m]$. *Then the leftmost occurrences of the distinct document identifiers in* ${\textsf{DA}}[\ell ..r]$ *are in the same positions as the values strictly less than* *m* *in* ${\textsf{ILCP}}[\ell ..r]$.

#### *Proof*

Let ${\textsf{SA}}_{S_j}[\ell _j..r_j]$ be the interval of all the suffixes of *S*_*j*_ starting with *P*[1..*m*]. Then ${\textsf{LCP}}_{S_j}[\ell _j] < m$, as otherwise $S_j[{\textsf{SA}}[\ell _j-1]..{\textsf{SA}}[\ell _j-1]+m-1] = S_j[{\textsf{SA}}[\ell _j]..{\textsf{SA}}[\ell _j]+m-1] = P$ as well, contradicting the definition of ℓ_*j*_. For the same reason, it holds that ${\textsf{LCP}}_{S_j}[\ell _j+k] \ge m$ for all $1 \le k \le r_j-\ell _j$.

Now let *S*_*j*_ start at position *p*_*j*_ + 1 in *T*, where $$p_j = |S_1 \cdots S_{j-1} |$$. Because each *S*_*j*_ is terminated by “$”, the lexicographic ordering between the suffixes *S*_*j*_[*k*..] in $${\textsf{SA}}_{S_j}$$ is the same as that of the corresponding suffixes *T*[*p*_*j*_ + *k*..] in $${\textsf{SA}}$$. Hence $$\langle {\textsf{SA}}[i] \mid {\textsf{DA}}[i] = j, 1\le i\le n \rangle = \langle p_j+{\textsf{SA}}_{S_j}[i] \mid 1\le i\le |S_j | \rangle$$. Or, put another way, $${\textsf{SA}}[i] = p_j + {\textsf{SA}}_{S_j}[{\textsf{rank}}_j({\textsf{DA}},i)]$$ whenever $${\textsf{DA}}[i]=j$$.

Now let *f*_*j*_ be the leftmost occurrence of *j* in ${\textsf{DA}}[\ell ..r]$. This means that ${\textsf{SA}}[f_j]$ is the lexicographically first suffix of *S*_*j*_ that starts with *P*. By the definition of ℓ_*j*_, it holds that $\ell _j = {\textsf{rank}}_j({\textsf{DA}},f_j)$. Thus, by definition of ${\textsf{ILCP}}$, it holds that ${\textsf{ILCP}}[f_j] = {\textsf{LCP}}_{S_j}[{\textsf{rank}}_j({\textsf{DA}},f_j)] = {\textsf{LCP}}_{S_j}[\ell _j] < m$, whereas all the other ${\textsf{ILCP}}[k]$ values, for $\ell \le k \le r$, where ${\textsf{DA}}[k]=j$, must be $\ge m$. $\square$

*Example* In the example above, if we search for $P[1..2]={\texttt {''TA''}}$, the resulting range is ${\textsf{SA}}[13..15]=\langle 8,3,1 \rangle$. The corresponding range ${\textsf{DA}}[13..15]=\langle 2,1,1\rangle$ indicates that the occurrence at ${\textsf{SA}}[13]$ is in *S*_2_ and those in ${\textsf{SA}}[14..15]$ are in *S*_1_. According to the lemma, it is sufficient to report the documents ${\textsf{DA}}[13]=2$ and ${\textsf{DA}}[14]=1$, as those are the positions in ${\textsf{ILCP}}[13..15] = \langle 0,0,2 \rangle$ with values less than |*P*| = 2.

Therefore, for the purposes of document listing, we can replace the *C* array by ${\textsf{ILCP}}$ in Muthukrishnan’s algorithm (Sect. 2.3): instead of recursing until we have listed all the positions *k* such that *C*[*k*] < ℓ, we recurse until we list all the positions *k* such that ${\textsf{ILCP}}[k] < m$. Instead of using it directly, however, we will design a variant that exploits repetitiveness in the string collection.

### ILCP on repetitive collections

The array ${\textsf{ILCP}}$ has yet another property, which makes it attractive for repetitive collections: it contains long runs of equal values. We give an analytic proof of this fact under a model where a base document *S* is generated at random under the very general A2 probabilistic model of Szpankowski (1993),^5^ and the collection is formed by performing some edits on *d* copies of *S*.

#### **Lemma 2**

*Let S[1..r]* *be a string generated under Szpankowski’s A2 model. Let* *T* *be formed by concatenating* *d* *copies of* *S*, *each terminated with the special symbol “$”, and then carrying out* *s* *edits (symbol insertions, deletions, or substitutions) at arbitrary positions in* *T* *(excluding the ‘$’s). Then, almost surely (a.s.* 6 *), the* $${\textsf{ILCP}}$$ *array of* *T* *is formed by* $$\rho \le r + {\mathcal {O}}\left( {s\lg (r+s)} \right)$$ *runs of equal values.*

#### *Proof*

Before applying the edit operations, we have $T = S_1 \cdots S_d$ and $S_j= {S\$}$ for all *j*. At this point, ${\textsf{ILCP}}$ is formed by at most *r* + 1 runs of equal values, since the *d* equal suffixes $S_j[{\textsf{SA}}_{S_j}[i]..r+1]$ must be contiguous in the suffix array ${\textsf{SA}}$ of *T*, in the area ${\textsf{SA}}[(i-1)d+1..id]$. Since the values $l={\textsf{LCP}}_{S_j}[i]$ are also equal, and ${\textsf{ILCP}}$ values are the ${\textsf{LCP}}_{S_j}$ values listed in the order of ${\textsf{SA}}$, it follows that ${\textsf{ILCP}}[(i-1)d+1..id]=l$ forms a run, and thus there are *r* + 1 = *n*/*d* runs in ${\textsf{ILCP}}$. Now, if we carry out *s* edit operations on *T*, any *S*_*j*_ will be of length at most *r* + *s* + 1. Consider an arbitrary edit operation at *T*[*k*]. It changes all the suffixes *T*[*k* − *h*..*n*] for all $0 \le h < k$. However, since a.s. the string depth of a leaf in the suffix tree of *S* is ${\mathcal {O}}\left( {\lg (r+s)} \right)$ (Szpankowski 1993), the suffix will possibly be moved in ${\textsf{SA}}$ only for $h = {\mathcal {O}}\left( {\lg (r+s)} \right)$. Thus, a.s., only ${\mathcal {O}}\left( {\lg (r+s)} \right)$ suffixes are moved in ${\textsf{SA}}$, and possibly the corresponding runs in ${\textsf{ILCP}}$ are broken. Hence $\rho \le r+{\mathcal {O}}\left( {s\lg (r+s)} \right)$ a.s. $\square$

Therefore, the number of runs depends linearly on the size of the base document and the number of edits, not on the total collection size. The proof generalizes the arguments of Mäkinen et al. (2010), which hold for uniformly distributed strings *S*. There is also experimental evidence (Mäkinen et al. 2010) that, in real-life text collections, a small change to a string usually causes only a small change to its ${\textsf{LCP}}$ array. Next we design a document listing data structure whose size is bounded in terms of *ρ*.

### Document listing

Let ${\textsf{LILCP}}[1..\rho ]$ be the array containing the partial sums of the lengths of the *ρ* runs in ${\textsf{ILCP}}$, and let ${{\textsf{VILCP}}}[1..\rho ]$ be the array containing the values in those runs. We can store ${{\textsf{LILCP}}}$ as a bitvector *L*[1..*n*] with *ρ* 1s, so that ${\textsf{LILCP}}[i]={\textsf{select}}(L,i)$. Then *L* can be stored using the structure of Okanohara and Sadakane (2007) that requires $\rho \lg (n/\rho )+{\mathcal {O}}\left( {\rho } \right)$ bits.

With this representation, it holds that ${\textsf{ILCP}}[i] = {\textsf{VILCP}}[{\textsf{rank}}_1(L,i)]$. We can map from any position *i* to its run $i'={\textsf{rank}}_1(L,i)$ in time ${\mathcal {O}}\left( {\lg (n/\rho )} \right)$, and from any run *i*′ to its starting position in ${\textsf{ILCP}}$, $i={\textsf{select}}(L,i')$, in constant time.

*Example* Consider the array ${\textsf{ILCP}}[1..15] = \langle 0,0,0,0,0,0,1,2,3,1,1,0,0,0,2\rangle$ of our running example. It has *ρ* = 7 runs, so we represent it with ${\textsf{VILCP}}[1..7]=\langle 0,1,2,3,1,0,2\rangle$ and $L[1..15] = 100000111101001$.

This is sufficient to emulate the document listing algorithm of Sadakane (2007) (Sect. 2.3) on a repetitive collection. We will use RLCSA as the ${\textsf{CSA}}$. The sparse bitvector *B*[1..*n*] marking the document beginnings in *T* will be represented in the same way as *L*, so that it requires $d\lg (n/d)+{\mathcal {O}}\left( {d} \right)$ bits and lets us compute any value ${\textsf{DA}}[i]={\textsf{rank}}_1(B,{\textsf{SA}}[i])$ in time ${\mathcal {O}}\left( {\textsf{lookup}\left( {n} \right) } \right)$. Finally, we build the compact RMQ data structure (Fischer and Heun 2011) on ${\textsf{VILCP}}$, requiring $2\rho +o(\rho )$ bits. We note that this RMQ structure does not need access to ${\textsf{VILCP}}$ to answer queries.

**Fig. 1:**
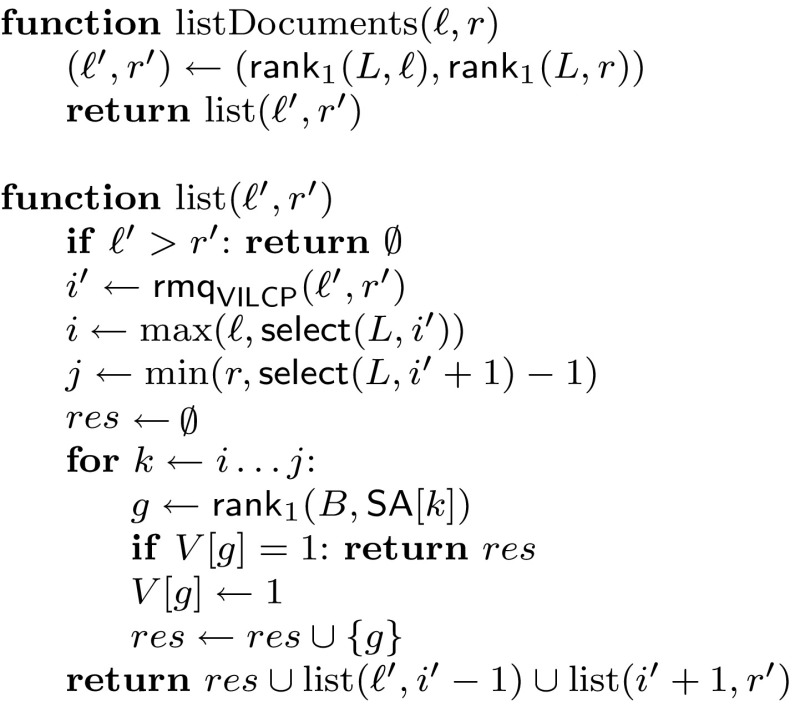
Pseudocode for document listing using the ${\textsf{ILCP}}$ array. Function listDocuments(ℓ, *r*) lists the documents from interval ${\textsf{SA}}[\ell ..r]$; ${\text {list}}(\ell ',r')$ returns the distinct documents mentioned in the runs ℓ′ to *r*′ that also belong to ${\textsf{DA}}[\ell ..r]$. We assume that in the beginning it holds *V*[*k*] = 0 for all *k*; this can be arranged by resetting to 0 the same positions after the query or by using initializable arrays. All the unions on *res* are known to be disjoint

Assume that we have already found the range ${\textsf{SA}}[\ell ..r]$ in ${\mathcal {O}}\left( {\textsf{search}\left( {m} \right) } \right)$ time. We compute $\ell ' = {\textsf{rank}}_1 (L,\ell )$ and $r' = {\textsf{rank}}_1 (L,r)$, which are the endpoints of the interval ${\textsf{VILCP}}[\ell '..r']$ containing the values in the runs in ${\textsf{ILCP}}[\ell ..r]$. Now we run Sadakane’s algorithm on ${\textsf{VILCP}}[\ell '..r']$. Each time we find a minimum at ${\textsf{VILCP}}[i']$, we remap it to the run ${\textsf{ILCP}}[i..j]$, where $i=\max (\ell ,{\textsf{select}}(L,i'))$ and $j=\min (r,{\textsf{select}}(L,i'+1)-1)$. For each $i \le k \le j$, we compute ${\textsf{DA}}[k]$ using *B* and RLCSA as explained, mark it in $V[{\textsf{DA}}[k]] \leftarrow 1$, and report it. If, however, it already holds that $V[{\textsf{DA}}[k]]=1$, we stop the recursion. Figure 1 gives the pseudocode.

We show next that this is correct as long as RMQ returns the leftmost minimum in the range and that we recurse first to the left and then to the right of each minimum ${\textsf{VILCP}}[i']$ found.

#### **Lemma 3**

*Using the procedure described, we correctly find all the positions* $\ell \le k \le r$ *such that* ${\textsf{ILCP}}[k] < m$.

#### *Proof*

Let $j={\textsf{DA}}[k]$ be the leftmost occurrence of document *j* in ${\textsf{DA}}[\ell ..r]$. By Lemma 1, among all the positions where ${\textsf{DA}}[k']=j$ in ${\textsf{DA}}[\ell ..r]$, *k* is the only one where ${\textsf{ILCP}}[k]<m$. Since we find a minimum ${\textsf{ILCP}}$ value in the range, and then explore the left subrange before the right subrange, it is not possible to find first another occurrence ${\textsf{DA}}[k']=j$, since it has a larger ${\textsf{ILCP}}$ value and is to the right of *k*. Therefore, when $V[{\textsf{DA}}[k]]=0$, that is, the first time we find a ${\textsf{DA}}[k]=j$, it must hold that ${\textsf{ILCP}}[k]<m$, and the same is true for all the other ${\textsf{ILCP}}$ values in the run. Hence it is correct to list all those documents and mark them in *V*. Conversely, whenever we find a $V[{\textsf{DA}}[k']]=1$, the document has already been reported. Thus this is not its leftmost occurrence and then ${\textsf{ILCP}}[k'] \ge m$ holds, as well as for the whole run. Hence it is correct to avoid reporting the whole run and to stop the recursion in the range, as the minimum value is already at least *m*. $\square$

Note that we are not storing ${\textsf{VILCP}}$ at all. We have obtained our first result for document listing, where we recall that *ρ* is small on repetitive collections (Lemma 2):

#### **Theorem 1**

*Let* $T=S_1 \cdot S_2 \cdots S_d$ *be the concatenation of* *d* *documents* *S*_*j*_, *and* ${\textsf{CSA}}$ *be a compressed suffix array on* *T*, *searching for any pattern P[1..m]* *in time* ${\textsf{search}}\left( {m} \right)$ *and accessing* ${\textsf{SA}}[i]$ *in time* ${\textsf{lookup}}\left( {n} \right)$. *Let* *ρ* *be the number of runs in the* ${\textsf{ILCP}}$ *array of* *T*. *We can store* *T* *in* $|{\textsf{CSA}} | + \rho \lg (n / \rho ) + {\mathcal {O}}\left( {\rho } \right) + d \lg (n / d) + {\mathcal {O}}\left( {d} \right) = |{\textsf{CSA}} | + {\mathcal {O}}\left( {(\rho +d)\lg n} \right)$ *bits such that document listing takes* ${\mathcal {O}}\left( {\textsf{search}\left( {m} \right) + {\textsf{df}}\cdot (\textsf{lookup}\left( {n} \right) +\lg n)} \right)$ *time*.

### Document counting

**Fig. 2:**
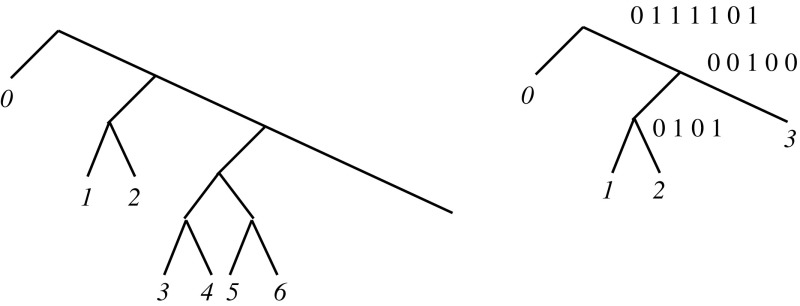
*On the left*, the schematic view of our skewed wavelet tree; *on the right*, the case of our running example where it represents ${\textsf{VILCP}}=\langle 0,1,2,3,1,0,2\rangle$

Array ${\textsf{ILCP}}$ also allows us to efficiently count the number of distinct documents where *P* appears, without listing them all. This time we will explicitly represent ${\textsf{VILCP}}$, in the following convenient way: consider a skewed wavelet tree (Sect. 2.2), where the leftmost leaf is at depth 1, the next 2 leaves are at depth 3, the next 4 leaves are at depth 5, and in general the 2^*d*−1^th to (2^*d*^ − 1)th leftmost leaves are at depth 2*d* − 1. Then the *i*th leftmost leaf is at depth $1+2\lfloor \lg i \rfloor = {\mathcal {O}}\left( {\lg i} \right)$. The number of wavelet tree nodes up to depth *d* is $\sum _{i=1}^{(d+1)/2} 2^i = 2(2^{(d+1)/2}-1)$. The number of nodes up to the depth of the *m*th leftmost leaf is maximized when *m* is of the form *m* = 2^*d*−1^, reaching $2(2^d-1)=4m-2={\mathcal {O}}\left( {m} \right)$. See Fig. 2.

Let *λ* be the maximum value in the ${\textsf{ILCP}}$ array. Then the height of the wavelet tree is ${\mathcal {O}}\left( {\lg \lambda } \right)$ and the representation of ${\textsf{VILCP}}$ takes at most $\rho \lg \lambda + o(\rho \lg \lambda )$ bits. If the documents *S* are generated using the A2 probabilistic model of Szpankowski (1993), then $\lambda ={\mathcal {O}}\left( {\lg |S |} \right) = {\mathcal {O}}\left( {\lg n} \right)$, and ${\textsf{VILCP}}$ uses $\rho \lg \lg n (1+o(1))$ bits. The same happens under the model used in Sect. 3.2.

The number of documents where *P* appears, ${\textsf{df}}$, is the number of times a value smaller than *m* occurs in ${\textsf{ILCP}}[\ell ..r]$. An algorithm to find all those values in a wavelet tree of ${\textsf{ILCP}}$ is as follows Gagie et al. (2012b). Start at the root with the range [ℓ..*r*] and its bitvector *W*. Go to the left child with the interval $[{\textsf{rank}}_0(W,\ell -1)+1..{\textsf{rank}}_0(W,r)]$ and to the right child with the interval $[{\textsf{rank}}_1(W,\ell -1)+1..{\textsf{rank}}_1(W,r)]$, stopping the recursion on empty intervals. This method arrives at all the wavelet tree leaves corresponding to the distinct values in ${\textsf{ILCP}}[\ell ..r]$. Moreover, if it arrives at a leaf *l* with interval ℓ_*l*_..*r*_*l*_, then there are r_*l*_ − ℓ_*l*_ + 1 occurrences of the symbol of that leaf in ${\textsf{ILCP}}[\ell ..r]$.

Now, in the skewed wavelet tree of ${\textsf{VILCP}}$, we are interested in the occurrences of symbols 0 to *m* − 1. Thus we apply the above algorithm but we do not enter into subtrees handling an interval of values that is disjoint with [0..*m* − 1]. Therefore, we only arrive at the *m* leftmost leaves of the wavelet tree, and thus traverse only ${\mathcal {O}}\left( {m} \right)$ wavelet tree nodes, in time ${\mathcal {O}}\left( {m} \right)$.

**Fig. 3:**
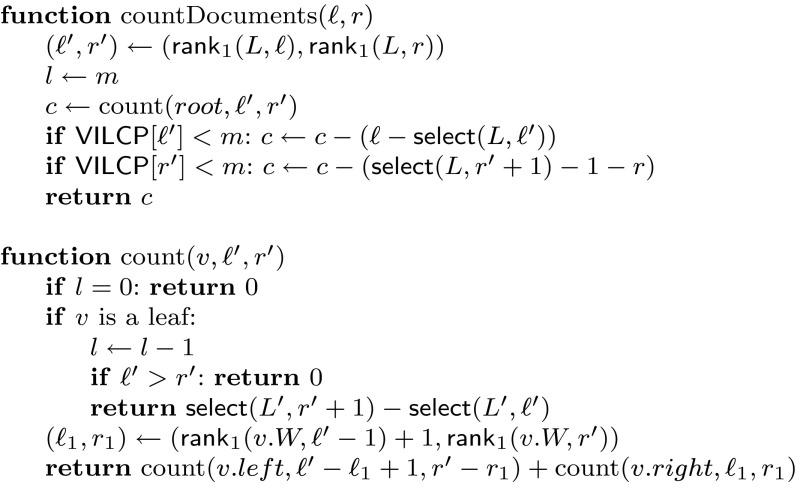
Document counting with the ${\textsf{ILCP}}$ array. Function countDocuments(ℓ, *r*) counts the distinct documents from interval ${\textsf{SA}}[\ell ..r]$; ${\text {count}}(v,\ell ',r')$ returns the number of documents mentioned in the runs ℓ′ to *r*′ under wavelet tree node *v* that also belong to ${\textsf{DA}}[\ell ..r]$. We assume that the wavelet tree root node is *root*, and that any internal wavelet tree node *v* has fields *v*.*W* (bitvector), *v*.*left* (left child), and *v*.*right* (right child). Global variable *l* is used to traverse the first *m* leaves. The access to ${\textsf{VILCP}}$ is also done with the wavelet tree

A complication is that ${\textsf{VILCP}}$ is the array of run length heads, so when we start at ${\textsf{VILCP}}[\ell '..r']$ and arrive at each leaf *l* with interval $[\ell '_l..r'_l]$, we only know that ${\textsf{VILCP}}[\ell '..r']$ contains from the $\ell '_l$th to the $r'_l$th occurrences of value *l* in ${\textsf{VILCP}}[\ell '..r']$. We store a reordering of the run lengths so that the runs corresponding to each value *l* are collected left to right in ${\textsf{ILCP}}$ and stored aligned to the wavelet tree leaf *l*. Those are concatenated into another bitmap $L'[1..n]$ with *ρ* 1s, similar to *L*, which allows us, using ${\textsf{select}}(L',\cdot )$, to count the total length spanned by the $\ell '_l$th to $r'_l$th runs in leaf *l*. By adding the areas spanned over the *m* leaves, we count the total number of documents where *P* occurs. Note that we need to correct the lengths of runs $\ell '$ and *r*′, as they may overlap the original interval ${\textsf{ILCP}}[\ell ..r]$. Figure 3 gives the pseudocode.

#### **Theorem 2**

*Let* $T=S_1 \cdot S_2 \cdots S_d$ *be the concatenation of* *d* *documents* *S*_*j*_, *and* ${\textsf{CSA}}$ *a compressed suffix array on* *T* *that searches for any pattern* *P*[1..*m*] *in time* ${\textsf{search}}\left( {m} \right)$. *Let* *ρ* *be the number of runs in the* ${\textsf{ILCP}}$ *array of* *T* *and* *λ* *be the maximum length of a repeated substring inside any* *S*_*j*_. *Then we can store* *T* *in* $|{\textsf{CSA}} | + \rho (\lg \lambda + 2\lg (n / \rho ) + {\mathcal {O}}\left( {1} \right) ) = |{\textsf{CSA}} |+{\mathcal {O}}\left( {\rho \lg n} \right)$ *bits such that the number of documents where a pattern* *P*[1..*m*] *occurs can be computed in time* ${\mathcal {O}}\left( {m+\textsf{search}\left( {m} \right) } \right)$.

## Precomputed document lists

In this section we introduce the idea of precomputing the answers of document retrieval queries for a sample of suffix tree nodes, and then exploit repetitiveness by grammar-compressing the resulting sets of answers. Such grammar compression is effective when the underlying collection is repetitive. The queries are then extremely fast on the sampled nodes, whereas on the others we have a way to bound the amount of work performed. The resulting structure is called PDL (Precomputed Document Lists), for which we develop a variant for document listing and another for top-*k* retrieval queries.

### Document listing

Let *v* be a suffix tree node. We write ${\textsf{SA}}_{v}$ to denote the interval of the suffix array covered by node *v*, and *D*_*v*_ to denote the set of distinct document identifiers occurring in the same interval of the document array. Given a block size *b* and a constant *β* ≥ 1, we build a sampled suffix tree that allows us to answer document listing queries efficiently. For any suffix tree node *v*, it holds that:

1. node *v* is sampled and thus set *D*_*v*_ is directly stored; or
2. $|{\textsf{SA}}_{v} | < b$, and thus documents can be listed in time ${\mathcal {O}}\left( {b \cdot \textsf{lookup}\left( {n} \right) } \right)$ by using a ${\textsf{CSA}}$ and the bitvectors *B* and *V* of Sect. 2.3; or
3. we can compute the set *D*_*v*_ as the union of stored sets $D_{u_{1}}, \ldots , D_{u_{k}}$ of total size at most $\beta \cdot |D_{v} |$, where nodes $u_{1}, \ldots , u_{k}$ are the children of *v* in the sampled suffix tree.

The purpose of rule 2 is to ensure that suffix array intervals solved by brute force are not longer than *b*. The purpose of rule 3 is to ensure that, if we have to rebuild an answer by merging a list of answers precomputed at descendant sampled suffix tree nodes, then the merging costs no more than *β* per result. That is, we can discard answers of nodes that are close to being the union of the answers of their descendant nodes, since we do not waste too much work in performing the unions of those descendants. Instead, if the answers of the descendants have many documents in common, then it is worth storing the answer at the node too; otherwise merging will require much work because the same document will be found many times (more than *β* on average).

We start by selecting suffix tree nodes $v_{1}, \ldots , v_{L}$, so that no selected node is an ancestor of another, and the intervals ${\textsf{SA}}_{v_{i}}$ of the selected nodes cover the entire suffix array. Given node *v* and its parent *w*, we select *v* if $|{\textsf{SA}}_{v} | \le b$ and $|{\textsf{SA}}_{w} | > b$, and store *D*_*v*_ with the node. These nodes *v* become the leaves of the sampled suffix tree, and we assume that they are numbered from left to right. We then assume that all the ancestors of those leaves belong to the sampled suffix tree, and proceed upward in the suffix tree removing some of them. Let *v* be an internal node, $u_{1}, \ldots , u_{k}$ its children, and *w* its parent. If the total size of sets $D_{u_{1}}, \ldots , D_{u_{k}}$ is at most $\beta \cdot |D_{v} |$, we remove node *v* from the tree, and add nodes $u_{1}, \ldots , u_{k}$ to the children of node *w*. Otherwise we keep node *v* in the sampled suffix tree, and store *D*_*v*_ there.

When the document collection is repetitive, the document array ${\textsf{DA}}[1..n]$ is also repetitive. This property has been used in the past to compress ${\textsf{DA}}$ using grammars (Navarro et al. 2014b). We can apply a similar idea on the *D*_*v*_ sets stored at the sampled suffix tree nodes, since *D*_*v*_ is a function of the range ${\textsf{DA}}[\ell ..r]$ that corresponds to node *v*.

Let $v_{1}, \ldots , v_{L}$ be the leaf nodes and $v_{L+1}, \ldots , v_{L+I}$ the internal nodes of the sampled suffix tree. We use grammar-based compression to replace frequent subsets in sets $D_{v_{1}}, \ldots , D_{v_{L+I}}$ with grammar rules expanding to those subsets. Given a set *Z* and a grammar rule *X* → *Y*, where $Y \subseteq \{ 1, \ldots , d \}$, we can replace *Z* with $(Z \cup \{ X \}) \setminus Y$, if $Y \subseteq Z$. As long as $|Y | \ge 2$ for all grammar rules *X* → *Y*, each set $D_{v_{i}}$ can be decompressed in ${\mathcal {O}}\left( {|D_{v_{i}} |} \right)$ time.

To choose the replacements, consider the bipartite graph with vertex sets $\{ v_{1}, \ldots , v_{L+I} \}$ and $\{ 1, \ldots , d \}$, with an edge from *v*_*i*_ to *j* if $j \in D_{v_{i}}$. Let *X* → *Y* be a grammar rule, and let *V* be the set of nodes *v*_*i*_ such that rule *X* → *Y* can be applied to set $D_{v_{i}}$. As $Y \subseteq D_{v_{i}}$ for all *v* ∈ *V*, the induced subgraph with vertex sets *V* and *Y* is a complete bipartite graph or a *biclique*. Many Web graph compression algorithms are based on finding bicliques or other dense subgraphs (Hernández and Navarro 2014), and we can use these algorithms to find a good grammar compressing the precomputed document lists.

When all rules have been applied, we store the reduced sets $D_{v_{1}}, \ldots , D_{v_{L+I}}$ as an array *A* of document and rule identifiers. The array takes $|A | \lg (d + n_{R})$ bits of space, where *n*_*R*_ is the total number of rules. We mark the first cell in the encoding of each set with a 1 in a bitvector *B*_*A*_[1..|*A*|], so that set $D_{v_{i}}$ can be retrieved by decompressing $A[{\textsf{select}}(B_{A}, i)..{\textsf{select}}(B_{A}, i+1) - 1]$. The bitvector takes |*A*|(1 + *o*(1) bits of space and answers ${\textsf{select}}$ queries in ${\mathcal {O}}\left( {1} \right)$ time. The grammar rules are stored similarly, in an array *G* taking $|G | \lg d$ bits, with a bitvector *B*_*G*_[1..|*G*|] of |*G*|(1 + *o*(1)) bits separating the array into rules (note that right hand sides of rules are formed only by terminals).

In addition to the sets and the grammar, we must also store the sampled suffix tree. A bitvector B _*L*_[1..*n*] marks the first cell of interval ${\textsf{SA}}_{v_{i}}$ for all leaf nodes *v*_*i*_, allowing us to convert interval ${\textsf{SA}}[\ell ..r]$ into a range of nodes $[ln..rn] = [{\textsf{rank}}_1(B_{L}, \ell ) .. {\textsf{rank}}_1(B_{L}, r+1) - 1]$. Using the format of Okanohara and Sadakane (2007) for *B*_*L*_, the bitvector takes $L \lg (n/L) + {\mathcal {O}}\left( {L} \right)$ bits, and answers ${\textsf{rank}}$ queries in ${\mathcal {O}}\left( {\lg (n/L)} \right)$ time and ${\textsf{select}}$ queries in constant time. A second bitvector *B*_*F*_[1..*L* + *I*], using (*L* + *I*)(1 + *o*(1)) bits and supporting ${\textsf{rank}}$ queries in constant time, marks the nodes that are the first children of their parents. An array *F*[1..*I*] of $I \lg I$ bits stores pointers from first children to their parent nodes, so that if node *v*_*i*_ is a first child, its parent node is *v*_*j*_, where $j = L + F[{\textsf{rank}}_1(B_{F}, i)]$. Finally, array *N*[1..*I*] of $I \lg L$ bits stores a pointer to the leaf node following those below each internal node.

**Fig. 4:**
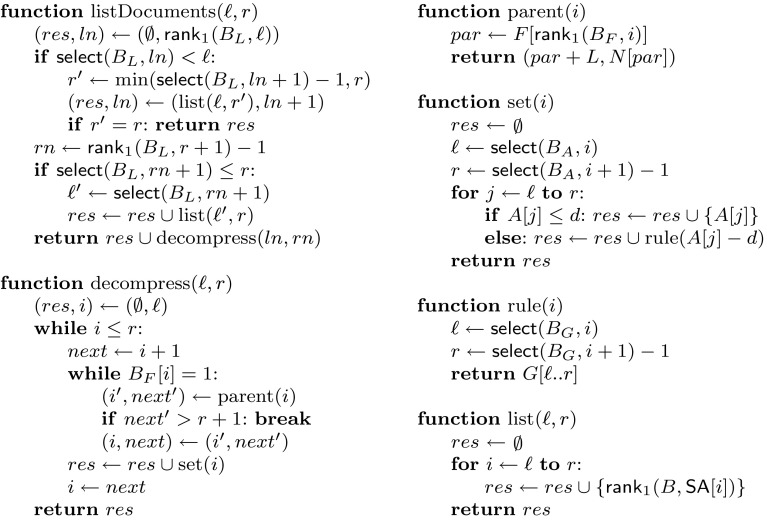
Document listing using precomputed answers. Function listDocuments(ℓ, *r*) lists the documents from interval ${\textsf{SA}}[\ell ..r]$; decompress(ℓ, *r*) decompresses the sets stored in nodes $v_{\ell }, \ldots , v_{r}$; parent(*i*) returns the parent node and the leaf node following it for a first child *v*_*i*_; set(*i*) decompresses the set stored in *v*_*i*_; rule(*i*) expands the *i*th grammar rule; and list(ℓ, *r*) lists the documents from interval ${\textsf{SA}}[\ell ..r]$ by using ${\textsf{CSA}}$ and bitvector *B*

Figure 4 gives the pseudocode for document listing using the precomputed answers. Function list(ℓ, *r*) takes ${\mathcal {O}}\left( {(r + 1 - \ell )\,\textsf{lookup}\left( {n} \right) } \right)$ time, set(*i*) takes ${\mathcal {O}}\left( {|D_{v_{i}} |} \right)$ time, and parent(*i*) takes ${\mathcal {O}}\left( {1} \right)$ time. Function decompress(ℓ, *r*) produces set *res* in time ${\mathcal {O}}\left( {|res |\cdot \beta h} \right)$, where *h* is the height of the sampled suffix tree: finding each set may take ${\mathcal {O}}\left( {h} \right)$ time, and we may encounter the same document ${\mathcal {O}}\left( {\beta } \right)$ times. Hence the total time for listDocuments(ℓ, *r*) is ${\mathcal {O}}\left( {{\textsf{df}}\cdot \beta h + \lg n} \right)$ for unions of precomputed answers, and ${\mathcal {O}}\left( {b \cdot \textsf{lookup}\left( {n} \right) } \right)$ otherwise. If the text follows the A2 model of Szpankowski (1993), then $h={\mathcal {O}}\left( {\lg n} \right)$ and the total time is on average ${\mathcal {O}}\left( {{\textsf{df}}\cdot \beta \lg n + b \cdot \textsf{lookup}\left( {n} \right) } \right)$.

We do not write the result as a theorem because we cannot upper bound the space used by the structure in terms of *b* and *β*. In a bad case like $T=a^{\ell -1}\$b^{\ell -1}\$c^{\ell -1}\$\ldots$, the suffix tree is formed by *d* long paths and the sampled suffix tree contains at least $d(n/d-b) = \varTheta (n)$ nodes (assuming *bd* = * o*(*n*)), so the total space is ${\mathcal {O}}\left( {n\lg n} \right)$ bits as in a classical suffix tree. In a good case, such as a balanced suffix tree (which also arises on texts following the A2 model), the sampled suffix tree has ${\mathcal {O}}\left( {n/b} \right)$ nodes. Although each such node *v* may store a list *D*_*v*_ with *b* entries, many of those entries are similar when the collection is repetitive, and thus their compression is effective.

### Top-*k* retrieval

Since we have the freedom to represent the documents in sets *D*_*v*_ in any order, we can in particular sort the document identifiers in decreasing order of their “frequencies”, that is, the number of times the string represented by *v* appears in the documents. Ties are broken by document identifiers in increasing order. Then a top-*k* query on a node *v* that stores its list *D*_*v*_ boils down to listing the first *k* elements of *D*_*v*_.

This time we cannot use the set-based grammar compressor, but we need, instead, a compressor that preserves the order. We use Re-Pair (Larsson and Moffat 2000), which produces a grammar where each nonterminal produces two new symbols, terminal or nonterminal. As Re-Pair decompression is recursive, decompression can be slower than in document listing, although it is still fast in practice and takes linear time in the length of the decompressed sequence.

In order to merge the results from multiple nodes in the sampled suffix tree, we need to store the frequency of each document. These are stored in the same order as the identifiers. Since the frequencies are nonincreasing, with potentially long runs of small values, we can represent them space-efficiently by run-length encoding the sequences and using differential encoding for the run heads. A node containing *s* suffixes in its subtree has at most ${\mathcal {O}}\left( {\sqrt{s}} \right)$ distinct frequencies, and the frequencies can be encoded in ${\mathcal {O}}\left( {\sqrt{s} \lg s} \right)$ bits.

There are two basic approaches to using the PDL structure for top-*k* document retrieval. First, we can store the document lists for all suffix tree nodes above the leaf blocks, producing a structure that is essentially an inverted index for all frequent substrings. This approach is very fast, as we need only decompress the first *k* document identifiers from the stored sequence, and it works well with repetitive collections thanks to the grammar-compression of the lists. Note that this enables *incremental top-k queries*, where value *k* is not given beforehand, but we extract documents with successively lower scores and can stop at any time. Note also that, in this version, it is not necessary to store the frequencies.

Alternatively, we can build the PDL structure as in Sect. 4.1, with some parameter *β*, to achieve better space usage. Answering queries will then be slower as we have to decompress multiple document sets, merge the sets, and determine the top *k* documents. We tried different heuristics for merging prefixes of the document sequences, stopping when a correct answer to the top-*k* query could be guaranteed. The heuristics did not generally work well, making brute-force merging the fastest alternative.

## Engineering a document counting structure

In this section we revisit a generic document counting structure by Sadakane (2007), which uses 2*n* + *o*(*n*) bits and answers counting queries in constant time. We show that the structure inherits the repetitiveness present in the text collection, which can then be exploited to reduce its space occupancy. Surprisingly, the structure also becomes repetitive with random and near-random data, such as unrelated DNA sequences, which is a result of interest for general string collections. We show how to take advantage of this redundancy in a number of different ways, leading to different time/space trade-offs.

### The basic bitvector

We describe the original document structure of Sadakane (2007), which computes ${\textsf{df}}$ in constant time given the locus of the pattern *P* (i.e., the suffix tree node arrived at when searching for *P*), while using just 2*n* + *o*(*n*) bits of space.

We start with the suffix tree of the text, and add new internal nodes to it to make it a binary tree. For each internal node *v* of the binary suffix tree, let *D*_*v*_ be again the set of distinct document identifiers in the corresponding range ${\textsf{DA}}[\ell ..r]$, and let ${\textsf{count}}(v)=|D_v|$ be the size of that set. If node *v* has children *u* and *w*, we define the number of *redundant* suffixes as $h(v) = |D_u \cap D_w |$. This allows us to compute ${\textsf{df}}$ recursively: ${\textsf{count}}(v) = {\textsf{count}}(u) + {\textsf{count}}(w) - h(v)$. By using the leaf nodes descending from *v*, [ℓ..r], as base cases, we can solve the recurrence:

$${\textsf{count}}(v) = {\textsf{count}}(\ell ,r) = (r + 1 - \ell ) - \sum _{u} h(u),$$

where the summation goes over the internal nodes of the subtree rooted at *v*.

We form an array *H*[1..*n* − 1] by traversing the internal nodes in inorder and listing the *h*(*v*) values. As the nodes are listed in inorder, subtrees form contiguous ranges in the array. We can therefore rewrite the solution as

$${\textsf{count}}(\ell ,r) = (r + 1 - \ell ) - \sum _{i=\ell }^{r-1} H[i].$$

To speed up the computation, we encode the array in unary as bitvector *H*′. Each cell *H*[*i*] is encoded as a 1-bit, followed by *H*[*i*] 0s. We can now compute the sum by counting the number of 0s between the 1s of ranks *ℓ* and *r*:

$${\textsf{count}}(\ell ,r) = 2(r - \ell ) - ({\textsf{select}}_{1}(H',r) - {\textsf{select}}_{1}(H',\ell )) + 1.$$

As there are *n* − 1 1s and *n* − *d* 0s, bitvector *H*′ takes at most 2*n* + *o*(*n*) bits.

### Compressing the bitvector

The original bitvector requires 2*n* + *o*(*n*) bits, regardless of the underlying data. This can be a considerable overhead with highly compressible collections, taking significantly more space than the ${\textsf{CSA}}$ (on top of which the structure operates). Fortunately, as we now show, the bitvector *H*′ used in Sadakane’s method is highly compressible. There are five main ways of compressing the bitvector, with different combinations of them working better with different datasets.

1. Let *V*_*v*_ be the set of nodes of the binary suffix tree corresponding to node *v* of the original suffix tree. As we only need to compute ${\textsf{count}}()$ for the nodes of the original suffix tree, the individual values of *h*(*u*), *u* ∈ *V*_*v*_, do not matter, as long as the sum $\sum _{u \in V_{v}} h(u)$ remains the same. We can therefore make bitvector *H*′ more compressible by setting $H[i] = \sum _{u \in V_{v}} h(u)$, where *i* is the inorder rank of node *v*, and *H*[*j*] = 0 for the rest of the nodes. As there are no real drawbacks in this reordering, we will use it with all of our variants of Sadakane’s method.
2. *Run-length encoding* works well with versioned collections and collections of random documents. When a pattern occurs in many documents, but no more than once in each, the corresponding subtree will be encoded as a run of 1s in *H*′.
3. When the documents in the collection have a versioned structure, we can reasonably expect *grammar compression* to be effective. To see this, consider a substring *x* that occurs in many documents, but at most once in each document. If each occurrence of substring *x* is preceded by symbol *a*, the subtrees of the binary suffix tree corresponding to patterns *x* and *ax* have an identical structure, and the corresponding areas in *D* are identical. Hence the subtrees are encoded identically in bitvector *H*′.
4. If the documents are internally repetitive but unrelated to each other, the suffix tree has many subtrees with suffixes from just one document. We can prune these subtrees into leaves in the binary suffix tree, using a *filter* bitvector *F*[1..*n* − 1] to mark the remaining nodes. Let *v* be a node of the binary suffix tree with inorder rank *i*. We will set* F*[*i*] = 1 iff ${\textsf{count}}(v) > 1$. Given a range [*ℓ*..*r* − 1] of nodes in the binary suffix tree, the corresponding subtree of the pruned tree is $[{\textsf{rank}}_{1}(F,\ell ) .. {\textsf{rank}}_{1}(F, r-1)]$. The filtered structure consists of bitvector *H*′ for the pruned tree and a compressed encoding of *F*.
5. We can also use filters based on the values in array *H* instead of the sizes of the document sets. If *H*[*i*] = 0 for most cells, we can use a *sparse filter* *F*_*S*_[1..*n* − 1], where *F*_*S*_[*i*] = 1 iff *H*[*i*] > 0, and build bitvector *H*′ only for those nodes. We can also encode positions with *H*[*i*] = 1 separately with a *1-filter* *F*_1_[1..*n* − 1], where *F*_1_[*i*] = 1 iff *H*[*i*] = 1. With a 1-filter, we do not write 0s in *H*′ for nodes with *H*[*i*] = 1, but instead subtract the number of 1s in *F*_1_[ℓ..*r* − 1] from the result of the query. It is also possible to use a sparse filter and a 1-filter simultaneously. In that case, we set *F*_*S*_[*i*] = 1 iff *H*[*i*] > 1.

### Analysis

We analyze the number of runs of 1s in bitvector *H*′ in the expected case. Assume that our document collection consists of *d* documents, each of length *r*, over an alphabet of size *σ*. We call string *S* *unique*, if it occurs at most once in every document. The subtree of the binary suffix tree corresponding to a unique string is encoded as a run of 1s in bitvector *H*′. If we can cover all leaves of the tree with *u* unique substrings, bitvector *H*′ has at most 2*u* runs of 1s.

**Fig. 5:**
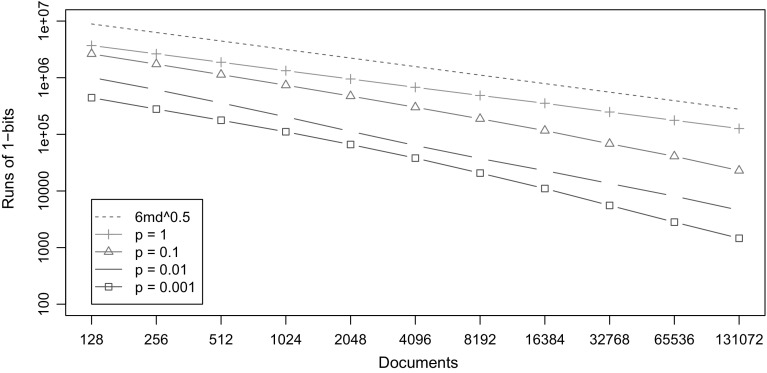
The number of runs of 1-bits in Sadakane’s bitvector *H*′ on synthetic collections of DNA sequences (*σ* = 4). Each collection has been generated by taking a random sequence of length *m* = 2^7^–2^17^, duplicating it *d* = 2^17^–2^7^ times (making the total size of the collection 2^24^), and mutating the sequences with random point mutations at probability *p* = 0.001–1. The mutations preserve zero-order empirical entropy by replacing the mutated symbol with a randomly chosen symbol according to the distribution in the original sequence. The *dashed line* represents the expected case upper bound for *p* = 1

Consider a random string of length *k*. Suppose the probability that the string occurs at least twice in a given document is at most $r^2 / (2 \sigma ^{2 k})$, which is the case if, e.g., we choose each document randomly or we choose one document randomly and generate the others by copying it and randomly substituting some symbols. By the union bound, the probability the string is non-unique is at most $d r^2 / (2 \sigma ^{2 k})$. Let *N*(*i*) be the number of non-unique strings of length $k_{i} = \lg _{\sigma } (r \sqrt{d}) + i$. As there are $\sigma ^{k_{i}}$ strings of length *k*_*i*_, the expected value of *N*(*i*) is at most $r \sqrt{d} / (2 \sigma ^{i})$. The expected size of the smallest cover of unique strings is therefore at most

$$(\sigma ^{k_{0}} - N(0)) + \sum _{i=1}^{\infty } (\sigma N(i-1) - N(i)) = r \sqrt{d} + (\sigma - 1) \sum _{i=0}^{\infty } N(i) \le \left( \frac{\sigma }{2} + 1 \right) r \sqrt{d},$$

where *σN*(*i* − 1) − *N*(*i*) is the number of strings that become unique at length *k*_*i*_. The number of runs of 1s in *H*′ is therefore sublinear in the size of the collection (*dr*). See Fig. 5 for an experimental confirmation of this analysis.

## A multi-term index

The queries we defined in the Introduction are *single-term*, that is, the query pattern *P* is a single string. In this section we show how our indexes for single-term retrieval can be used for *ranked multi-term* queries on repetitive text collections. The key idea is to regard our incremental top-*k* algorithm of Sect. 4.2 as an *abstract representation* of the inverted lists of the individual query terms, sorted by decreasing weight, and then apply any algorithm that traverses those lists sequentially. Since our relevance score will depend on the term frequency and the document frequency of the terms, we will integrate a document counting structure as well (Sects. 3.4 or 5).

Let $Q = \langle q_{1}, \dots , q_{m} \rangle$ be a query consisting of *m* patterns *q*_*i*_. We support ranked queries, which return the *k* documents with the highest scores among the documents matching the query. A *disjunctive* or *ranked-OR* query matches document *D* if at least one of the patterns occurs in it, while a *conjunctive* or *ranked-AND* query matches *D* if all query patterns occur in it. Our index supports both conjunctive and disjunctive queries with ${\textsf{tf}}{\textsf{-}}{\textsf{idf}}$-like scores

$$w(D, Q) = \sum _{i=1}^{m} w(D, q_{i}) = \sum _{i=1}^{m} f({\textsf{tf}}(D, q_{i})) \cdot g({\textsf{df}}(q_{i})),$$

where *f* ≥ 0 is an increasing function, ${\textsf{tf}}(D, q_{i})$ is the *term frequency* (the number of occurrences) of pattern *q*_*i*_ in document *D*, *g* ≥ 0 is a decreasing function, and ${\textsf{df}}(q_{i})$ is the *document frequency* of pattern *q*_*i*_. For example, the standard ${\textsf{tf}}{\textsf{-}}{\textsf{idf}}$ scoring scheme corresponds to using $f({\textsf{tf}}) = {\textsf{tf}}$ and $g({\textsf{df}}) = \lg (d / \max ({\textsf{df}}, 1))$.

From Sect. 4.2, we use the incremental variant, which stores the full answers for all the suffix tree nodes above leaves. The query algorithm uses ${\textsf{CSA}}$ to find the lexicographic range [ℓ_*i*_..*r*_*i*_] matching each pattern *q*_*i*_. We then use PDL to find the sparse suffix tree node *v*_*i*_ corresponding to range [ℓ_*i*_..*r*_*i*_] and fetch its list $D_{v_i}$, which is stored in decreasing term frequency order. If *v*_*i*_ is not in the sparse suffix tree, we use instead the ${\textsf{CSA}}$ to build $D_{v_i}$ by brute force from ${\textsf{SA}}[\ell _{i}..r_{i}]$. We also compute ${\textsf{df}}(q_{i})={\textsf{count}}(v_i)$ for all query patterns *q*_*i*_ with our document counting structure. The algorithm then iterates the following loop with $k' = 2k, 4k, 8k, \ldots$:

1. Extract *k*′ more documents from the document list of *v*_*i*_ for each pattern *q*_*i*_.
2. If the query is conjunctive, filter out extracted documents that do not match the query patterns with completely decompressed document lists.
3. Determine a lower bound for *w*(*D*, *Q*) for all documents *D* extracted so far. If document *D* has not been encountered in the document list of *v*_*i*_, use 0 as a lower bound for *w*(*D*, *q*_*i*_).
4. Determine an upper bound for *w*(*D*, *Q*) for all documents *D*. If document *D* has not been encountered in the document list of *v*_*i*_, use ${\textsf{tf}}(D', q_{i})$, where *D*′ is the next unextracted document for pattern *q*_*i*_, as an upper bound for ${\textsf{tf}}(D, q_{i})$.
5. If the query is disjunctive, filter out extracted documents *D* with smaller upper bounds for *w*(*D*, *Q*) than the lower bounds for the current top-*k* documents. Stop if the top-*k* set cannot change further.
6. If the query is conjunctive, stop if the top-*k* documents match all query patterns and the upper bounds for the remaining documents are lower than the lower bounds for the top-*k* documents.

The algorithm always finds a correct top-*k* set, although the scores may be incorrect if a disjunctive query stops early.

## Experiments and discussion

### Experimental setup

#### Document collections

We performed extensive experiments with both real and synthetic collections.^7^ Most of our document collections were relatively small, around 100 MB in size, as some of the implementations (Navarro et al. 2014b) use 32-bit libraries. We also used larger versions of some collections, up to 1 GB in size, to see how the collection size affects the results. In general, collection size is more important in top-*k* document retrieval. Increasing the number of documents generally increases the ${\textsf{df}}/k$ ratio, and thus makes brute-force solutions based on document listing less appealing. In document listing, the size of the documents is more important than collection size, as a large $occ /{\textsf{df}}$ ratio makes brute-force solutions based on pattern matching less appealing.

The performance of various solutions depends both on the repetitiveness of the collection and the type of the repetitiveness. Hence we used a fair number of real and synthetic collections with different characteristics for our experiments. We describe them next, and summarize their statistics in Table 2.

**Table 2:** Statistics for document collections (small, medium, and large variants)

Collection	Size	CSA size	Documents	Avg. doc size	Patterns	Occurrences	Document occs	Occs per doc
	(*n*) (MB)	(RLCSA) (MB)	(*d*)	(*n* / *d*)		($\overline{ occ }$)	($\overline{ {\textsf{df}} }$)	($\overline{ occ /{\textsf{df}} }$)
**Page**	110	2.58	60	1,919,382	7658	781	3	242.75
	641	9.00	190	3,534,921	14,286	2601	6	444.79
	1037	17.45	280	3,883,145	20,536	2889	7	429.04
**Revision**	110	2.59	8834	13,005	7658	776	371	2.09
	640	9.04	31,208	21,490	14,284	2592	1065	2.43
	1035	17.55	65,565	16,552	20,536	2876	1188	2.42
**Enwiki**	113	49.44	7000	16,932	18,935	1904	505	3.77
	639	309.31	44,000	15,236	19,628	10,316	2856	3.61
	1034	482.16	90,000	12,050	19,805	17,092	4976	3.44
**Influenza**	137	5.52	100,000	1436	1000	24,975	18,547	1.35
	321	10.53	227,356	1480	1000	59,997	44,012	1.36
**Swissprot**	54	25.19	143,244	398	10,000	160	121	1.33
**Wiki**	1432	42.90	103,190	14,540				
**DNA**	95		100,000		889–1000			
**Concat**	95		10–1000		7538–15,272			
**Version**	95		10,000		7537–15,271			

Collection size, RLCSA size without suffix array samples, number of documents, average document length, number of patterns, average number of occurrences and document occurrences, and the ratio of occurrences to document occurrences. For the synthetic collections (**DNA**, **Concat**, and **Version**), most of the statistics vary greatly

*A note on collection size* The index structures evaluated in this paper should be understood as promising algorithmic ideas. In most implementations, the construction algorithms do not scale up for collections larger than a couple of gigabytes. This is often intentional. In this line of research, being able to easily evaluate variations of the fundamental idea is more important than the speed or memory usage of construction. As a result, many of the construction algorithms build an explicit suffix tree for the collection and store various kinds of additional information in the nodes. Better construction algorithms can be designed once the most promising ideas have been identified. See “Appendix 2” for further discussion on index construction.

*Real collections* We use various document collections from real-life repetitive scenarios. Some collections come in small, medium, and large variants. **Page** and **Revision** are repetitive collections generated from a Finnish-language Wikipedia archive with full version history. There are 60 (small), 190 (medium), or 280 (large) pages with a total of 8834, 31,208, or 65,565 revisions. In **Page**, all the revisions of a page form a single document, while each revision becomes a separate document in **Revision**. **Enwiki** is a non-repetitive collection of 7000, 44,000, or 90,000 pages from a snapshot of the English-language Wikipedia. **Influenza** is a repetitive collection containing 100,000 or 227,356 sequences from influenza virus genomes (we only have small and large variants). **Swissprot** is a non-repetitive collection of 143,244 protein sequences used in many document retrieval papers (e.g., Navarro et al. 2014b). As the full collection is only 54 MB, only the small version of **Swissprot** exists. **Wiki** is a repetitive collection similar to **Revision**. It is generated by sampling all revisions of 1% of pages from the English-language versions of Wikibooks, Wikinews, Wikiquote, and Wikivoyage.

*Synthetic collections* To explore the effect of collection repetitiveness on document retrieval performance in more detail, we generated three types of synthetic collections, using files from the Pizza & Chili corpus.^8^ **DNA** is similar to **Influenza**. Each collection has *d* = 1, 10, 100, or 1000 base documents, 100,000/*d* variants of each base document, and mutation rate *p* = 0.001, 0.003, 0.01, 0.03, or 0.1. We take a prefix of length 1000 from the Pizza & Chili DNA file and generate the base documents by mutating the prefix at probability 10*p* under the same model as in Fig. 5. We then generate the variants in the same way with mutation rate *p*. **Concat** and **Version** are similar to **Page** and **Revision**, respectively. We read *d* = 10, 100, or 1000 base documents of length 10,000 from the Pizza & Chili English file, and generate 10,000/*d* variants of each base document with mutation rates 0.001, 0.003, 0.01, 0.03, and 0.1, as above. Each variant becomes a separate document in **Version**, while all variants of the same base document are concatenated into a single document in **Concat**.

#### Queries

*Real collections* For **Page** and **Revision**, we downloaded a list of Finnish words from the Institute for the Languages in Finland, and chose all words of length ≥5 that occur in the collection. For **Enwiki**, we used search terms from an MSN query log with stopwords filtered out. We generated 20,000 patterns according to term frequencies, and selected those that occur in the collection. For **Influenza**, we extracted 100,000 random substrings of length 7, filtered out duplicates, and kept the 1000 patterns with the largest $occ /{\textsf{df}}$ ratios. For **Swissprot**, we extracted 200,000 random substrings of length 5, filtered out duplicates, and kept the 10,000 patterns with the largest $occ /{\textsf{df}}$ ratios. For **Wiki**, we used the TREC 2006 Terabyte Track efficiency queries^9^ consisting of 411,394 terms in 100,000 queries.

*Synthetic collections* We generated the patterns for **DNA** with a similar process as for **Influenza** and **Swissprot**. We extracted 100,000 substrings of length 7, filtered out duplicates, and chose the 1000 with the largest $occ /{\textsf{df}}$ ratios. For **Concat** and **Version**, patterns were generated from the MSN query log in the same way as for **Enwiki**.

#### Test environment

We used two separate systems for the experiments. For document listing and document counting, our test environment had two 2.40 GHz quad-core Intel Xeon E5620 processors and 96 GB memory. Only one core was used for the queries. The operating system was Ubuntu 12.04 with Linux kernel 3.2.0. All code was written in C++. We used g++ version 4.6.3 for the document listing experiments and version 4.8.1 for the document counting experiments.

For the top-*k* retrieval and ${\textsf{tf}}{\textsf{-}}{\textsf{idf}}$ experiments, we used another system with two 16-core AMD Opteron 6378 processors and 256 GB memory. We used only a single core for the single-term queries and up to 32 cores for the multi-term queries. The operating system was Ubuntu 12.04 with Linux kernel 3.2.0. All code was written in C++ and compiled with g++ version 4.9.2.

We executed the query benchmarks in the following way:

1. Load the RLCSA with the desired sample period for the current collection into memory.
2. Load the query patterns corresponding to the collection into memory and execute ${\textsf{find}}$ queries in the RLCSA. Store the resulting lexicographic ranges [ℓ..*r*] in vector *V*.
3. Load the index to be benchmarked into memory.
4. Iterate through vector *V* once using a single thread and execute the desired query for each range [ℓ..*r*]. Measure the total wall clock time for executing the queries.

We divided the measured time by the number of patterns, and listed the average time per query in milliseconds or microseconds and the size of the index structure in bits per symbol. There were certain exceptions:

- **LZ** and **Grammar** do not use a ${\textsf{CSA}}$. With them, we iterated through the vector of patterns as in step 4, once the index and the patterns had been loaded into memory. The average time required to get the range [ℓ..*r*] in ${\textsf{CSA}}$-based indexes (4−6 μs, depending on the collection) was negligible compared to the average query times of **LZ** (at least 170 μs) and **Grammar** (at least 760 μs).
- We used the existing benchmark code with **SURF**. The code first loads the index into memory and then iterates through the pattern file by reading one line at a time. To reduce the overhead from reading the patterns, we cached them by using cat> /dev/null. Because **SURF** queries were based on the pattern instead of the corresponding range [ℓ..*r*], we executed ${\textsf{find}}$ queries first and subtracted the time used for them from the subsequent top-*k* queries.
- In our ${\textsf{tf}}{\textsf{-}}{\textsf{idf}}$ index, we parallelized step 4 using the OpenMP parallel for construct.
- We used the existing benchmark code with Terrier. We cached the queries as with **SURF**, set trec.querying.outputformat to NullOutputFormat, and set the logging level to off.

### Document listing

We compare our new proposals from Sects. 3.3 and 4.1 to the existing document listing solutions. We also aim to determine when these sophisticated approaches are better than brute-force solutions based on pattern matching.

#### Indexes

*Brute force* (**Brute**) These algorithms simply sort the document identifiers in the range ${\textsf{DA}}[\ell ..r]$ and report each of them once. **Brute-D** stores ${\textsf{DA}}$ in $n \lg d$ bits, while **Brute-L** retrieves the range ${\textsf{SA}}[\ell ..r]$ with the ${\textsf{locate}}$ functionality of the ${\textsf{CSA}}$ and uses bitvector *B* to convert it to ${\textsf{DA}}[\ell ..r]$.

*Sadakane* (**Sada**) This family of algorithms is based on the improvements of Sadakane (2007) to the algorithm of Muthukrishnan (2002). **Sada-L** is the original algorithm, while **Sada-D** uses an explicit document array ${\textsf{DA}}$ instead of retrieving the document identifiers with ${\textsf{locate}}$.

*ILCP* (**ILCP**) This is our proposal in Sect. 3.3. The algorithms are the same as those of Sadakane (2007), but they run on the run-length encoded ${\textsf{ILCP}}$ array. As for **Sada**, **ILCP-L** obtains the document identifiers using ${\textsf{locate}}$ on the ${\textsf{CSA}}$, whereas **ILCP-D** stores array ${\textsf{DA}}$ explicitly.

*Wavelet tree* (**WT**) This index stores the document array in a wavelet tree (Sect. 2.2) to efficiently find the distinct elements in ${\textsf{DA}}[\ell ..r]$ (Välimäki and Mäkinen 2007). The best known implementation of this idea (Navarro et al. 2014b) uses plain, entropy-compressed, and grammar-compressed bitvectors in the wavelet tree-depending on the level. Our **WT** implementation uses a heuristic similar to the original WT-alpha (Navarro et al. 2014b), multiplying the size of the plain bitvector by 0.81 and the size of the entropy-compressed bitvector by 0.9, before choosing the smallest one for each level of the tree. These constants were determined by experimental tuning.

*Precomputed document lists* (**PDL**) This is our proposal in Sect. 4.1. Our implementation resorts to **Brute-L** to handle the short regions that the index does not cover. The variant **PDL-BC** compresses sets of equal documents using a Web graph compressor (Hernández and Navarro 2014). **PDL-RP** uses Re-Pair compression (Larsson and Moffat 2000) as implemented by Navarro^10^ and stores the dictionary in plain form. We use block size *b* = 256 and storing factor *β* = 16, which have proved to be good general-purpose parameter values.

*Grammar-based* (**Grammar**) This index (Claude and Munro 2013) is an adaptation of a grammar-compressed self-index (Claude and Navarro 2012) to document listing. Conceptually similar to **PDL**, **Grammar** uses Re-Pair to parse the collection. For each nonterminal symbol in the grammar, it stores the set of identifiers of the documents whose encoding contains the symbol. A second round of Re-Pair is used to compress the sets. Unlike most of the other solutions, **Grammar** is an independent index and needs no ${\textsf{CSA}}$ to operate.

*Lempel-Ziv* (**LZ**) This index (Ferrada and Navarro 2013) is an adaptation of a pattern-matching index based on LZ78 parsing (Navarro 2004) to document listing. Like **Grammar**, **LZ** does not need a ${\textsf{CSA}}$.

We implemented **Brute**, **Sada**, ${\textsf{ILCP}}$, and the **PDL** variants ourselves^11^ and modified existing implementations of **WT**, **Grammar**, and **LZ** for our purposes. We always used the RLCSA (Mäkinen et al. 2010) as the ${\textsf{CSA}}$, as it performs well on repetitive collections. The ${\textsf{locate}}$ support in RLCSA includes optimizations for long query ranges and repetitive collections, which is important for **Brute-L** and **ILCP-L**. We used suffix array sample periods 8, 16, 32, 64, 128 for non-repetitive collections and 32, 64, 128, 256, 512 for repetitive ones.

When a document listing solution uses a ${\textsf{CSA}}$, we start the queries from the lexicographic range [ℓ..*r*] instead of the pattern *P*. This allows us to see the performance differences between the fastest solutions better. The average time required for obtaining the ranges was 4–6 μs per pattern, depending on the collection, which is negligible compared to the average time used by **Grammar** (at least 760 μs) and **LZ** (at least 170 μs).

#### Results

**Fig. 6:**
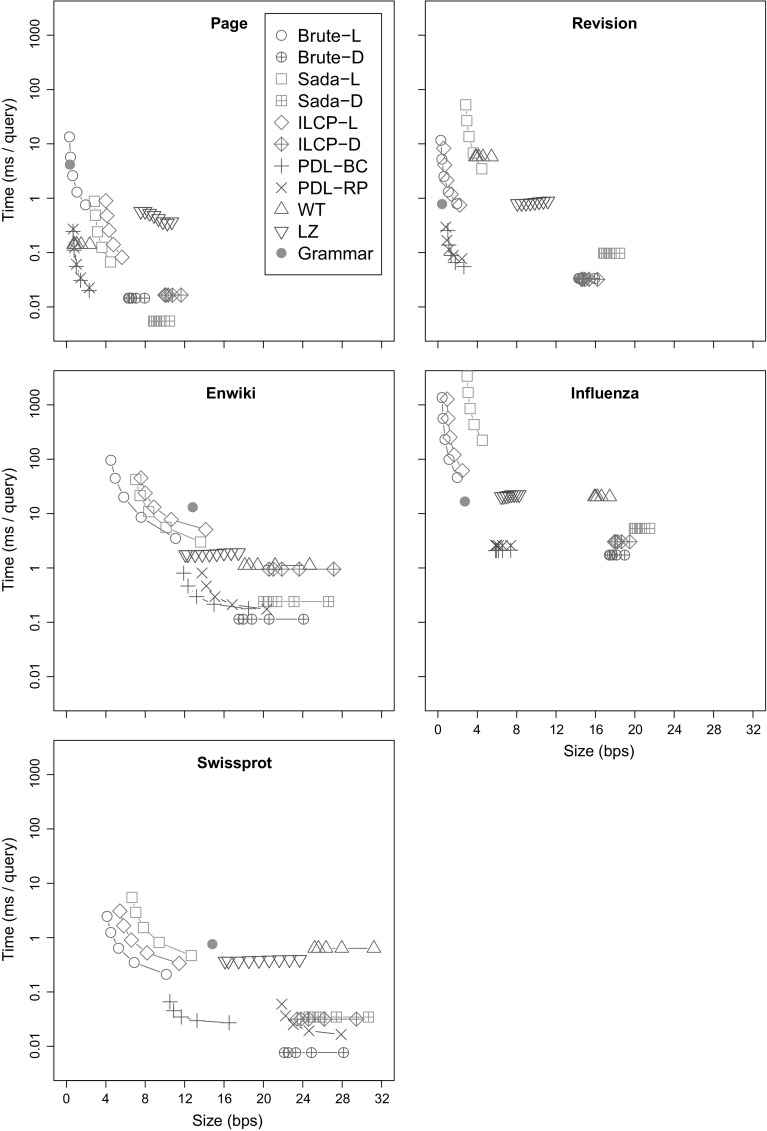
Document listing on small real collections. The total size of the index in bits per symbol (*x*) and the average time per query in milliseconds (*y*)

**Fig. 7:**
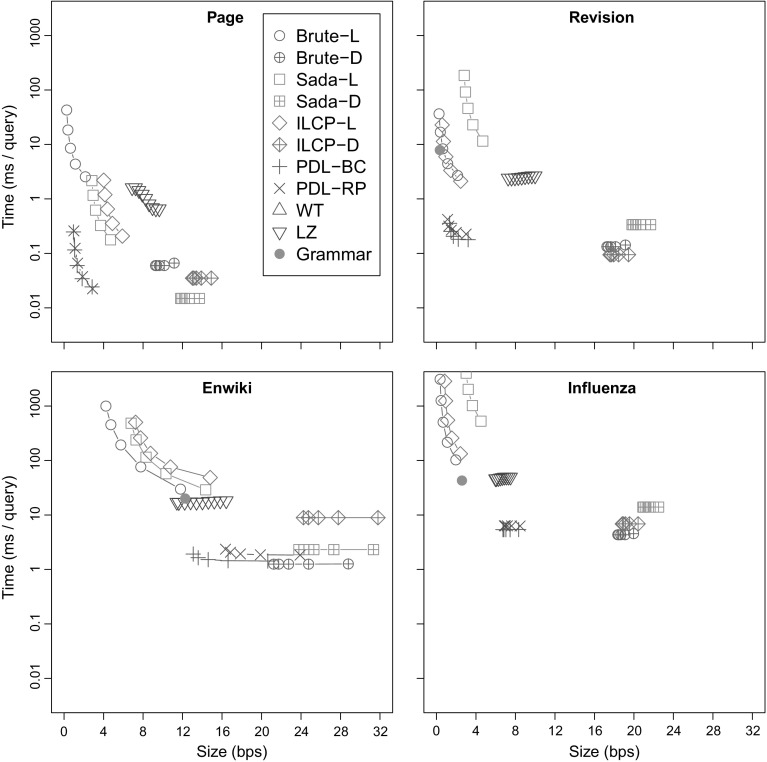
Document listing on large real collections. The total size of the index in bits per symbol (*x*) and the average time per query in milliseconds (*y*)

*Real collections* Figures 6 and 7 contain the results for document listing with small and large real collections, respectively. For most of the indexes, the time/space trade-off is given by the RLCSA sample period. The trade-off of **LZ** comes from a parameter specific to that structure involving RMQs (Ferrada and Navarro 2013). **Grammar** has no trade-off.

**Brute-L** always uses the least amount of space, but it is also the slowest solution. In collections with many short documents (i.e., all except **Page**), we have $occ /{\textsf{df}}< 4$ on the average. The additional effort made by **Sada-L** and **ILCP-L** to report each document only once does not pay off, and the space used by the RMQ structure is better spent on increasing the number of suffix array samples for **Brute-L**. The difference is, however, very noticeable on **Page**, where the documents are large and there are hundreds of occurrences of the pattern in each document. **ILCP-L** uses less space than **Sada-L** when the collection is repetitive and contains many similar documents (i.e., on **Revision** and **Influenza**); otherwise **Sada-L** is slightly smaller.

The two **PDL** alternatives usually achieve similar performance, but in some cases **PDL-BC** uses much less space. **PDL-BC**, in turn, can use significantly more space than **Brute-L**, **Sada-L**, and **ILCP-L**, but is always orders of magnitude faster. The document sets of versioned collections such as **Page** and **Revision** are very compressible, making the collections very suitable for **PDL**. On the other hand, grammar-based compression cannot reduce the size of the stored document sets enough when the collections are non-repetitive. Repetitive but unstructured collections like **Influenza** represent an interesting special case. When the number of revisions of each base document is much larger than the block size *b*, each leaf block stores an essentially random subset of the revisions, which cannot be compressed very well.

Among the other indexes, **Sada-D** and **ILCP-D** can be significantly faster than **PDL-BC**, but they also use much more space. From the non-${\textsf{CSA}}$-based indexes, **Grammar** reaches the Pareto-optimal curve on **Revision** and **Influenza**, while being too slow or too large on the other collections. We did not build **Grammar** for the large version of **Page**, as it would have taken several months.

In general, we can recommend **PDL-BC** as a medium-space alternative for document listing. When less space is available, we can use **ILCP-L**, which offers robust time and space guarantees. If the documents are small, we can even use **Brute-L**. Further, we can use fast document counting to compare ${\textsf{df}}$ with *occ* = *r* − ℓ + 1, and choose between **ILCP-L** and **Brute-L** according to the results.

**Fig. 8:**
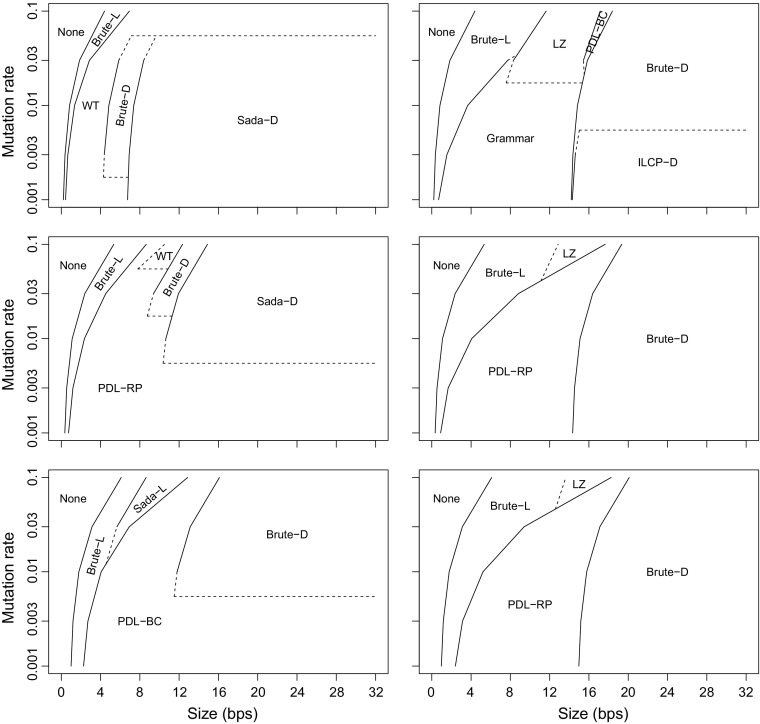
Document listing on synthetic collections. The fastest solution for a given size in bits per symbol and a mutation rate. From *top* to *bottom*: 10, 100, and 1000 base documents with **Concat** (*left*) and **Version** (*right*). **None** denotes that no solution can achieve that size

**Fig. 9:**
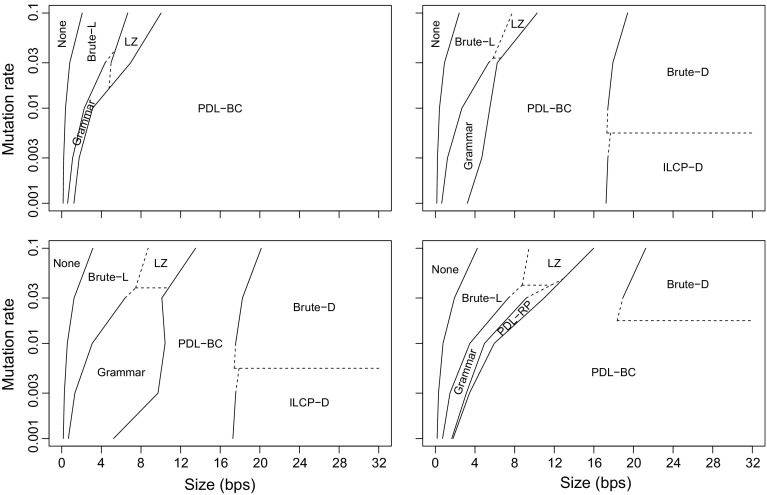
Document listing on synthetic collections. The fastest solution for a given size in bits per symbol and a mutation rate. **DNA** with 1 (*top left*), 10 (*top right*), 100 (*bottom left*), and 1000 (*bottom right*) base documents. **None** denotes that no solution can achieve that size

*Synthetic collections* Figures 8 and 9 show our document listing results with synthetic collections. Due to the large number of collections, the results for a given collection type and number of base documents are combined in a single plot, showing the fastest algorithm for a given amount of space and mutation rate. Solid lines connect measurements that are the fastest for their size, while dashed lines are rough interpolations.

The plots were simplified in two ways. Algorithms providing a marginal and/or inconsistent improvement in speed in a very narrow region (mainly **Sada-L** and **ILCP-L**) were left out. When **PDL-BC** and **PDL-RP** had a very similar performance, only one of them was chosen for the plot.

On **DNA**, **Grammar** was a good solution for small mutation rates, while **LZ** was good with larger mutation rates. With more space available, **PDL-BC** became the fastest algorithm. **Brute-D** and **ILCP-D** were often slightly faster than **PDL**, when there was enough space available to store the document array. On **Concat** and **Version**, **PDL** was usually a good mid-range solution, with **PDL-RP** being usually smaller than **PDL-BC**. The exceptions were the collections with 10 base documents, where the number of variants (1000) was clearly larger than the block size (256). With no other structure in the collection, **PDL** was unable to find a good grammar to compress the sets. At the large end of the size scale, algorithms using an explicit document array ${\textsf{DA}}$ were usually the fastest choices.

### Top-*k* retrieval

#### Indexes

We compare the following top-*k* retrieval algorithms. Many of them share names with the corresponding document listing structures described in Sect. 7.2.1.

*Brute force* (**Brute**) These algorithms correspond to the document listing algorithms **Brute-D** and **Brute-L**. To perform top-*k* retrieval, we not only collect the distinct document identifiers after sorting ${\textsf{DA}}[\ell ..r]$, we also record the number of times each one appears. The *k* identifiers appearing most frequently are then reported.

*Precomputed document lists* (**PDL**) We use the variant of **PDL-RP** modified for top-*k* retrieval, as described in Sect. 4.2. **PDL-** *b* denotes PDL with block size *b* and with document sets for all suffix tree nodes above the leaf blocks, while **PDL-** *b* **+F** is the same with term frequencies. **PDL-** *b*-*β* is PDL with block size *b* and storing factor *β*.

*Large and fast* (**SURF**) This index (Gog and Navarro 2015b) is based on a conceptual idea by Navarro and Nekrich (2012), and improves upon a previous implementation (Konow and Navarro 2013). It can answer top-*k* queries quickly if the pattern occurs at least twice in each reported document. If documents with just one occurrence are needed, **SURF** uses a variant of **Sada-L** to find them.

We implemented the **Brute** and **PDL** variants ourselves^12^ and used the existing implementation of **SURF**.^13^ While **WT** (Navarro et al. 2014b) also supports top-*k* queries, the 32-bit implementation cannot index the large versions of the document collections used in the experiments. As with document listing, we subtracted the time required for finding the lexicographic ranges [ℓ..*r*] using a ${\textsf{CSA}}$ from the measured query times. **SURF** uses a ${\textsf{CSA}}$ from the SDSL library (Gog et al. 2014), while the rest of the indexes use RLCSA.

#### Results

**Fig. 10:**
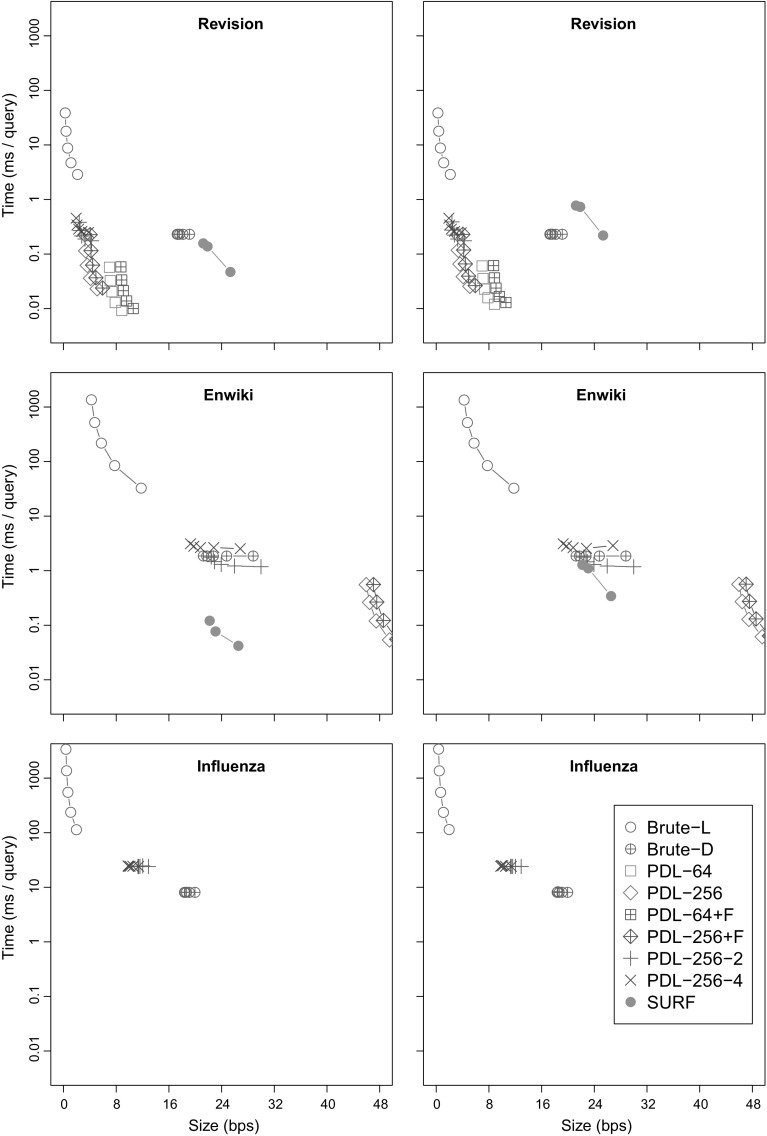
Single-term top-*k* retrieval on real collections with *k* = 10 (*left*) and *k* = 100 (*right*). The total size of the index in bits per symbol (x) and the average time per query in milliseconds (y)

Figure 10 contains the results for top-*k* retrieval using the large versions of the real collections. We left **Page** out of the results, as the number of documents (280) was too low for meaningful top-*k* queries. For most of the indexes, the time/space trade-off is given by the RLCSA sample period, while the results for **SURF** are for the three variants presented in the paper.

The three collections proved to be very different. With **Revision**, the **PDL** variants were both fast and space-efficient. When storing factor *β* was not set, the total query times were dominated by rare patterns, for which **PDL** had to resort to using **Brute-L**. This also made block size *b* an important time/space trade-off. When the storing factor was set, the index became smaller and slower and the trade-offs became less significant. **SURF** was larger and faster than **Brute-D** with *k* = 10 but became slow with *k* = 100.

On Enwiki, the variants of **PDL** with storing factor *β* set had a performance similar to **Brute-D**. **SURF** was faster with roughly the same space usage. **PDL** with no storing factor was much larger than the other solutions. However, its time performance became competitive for *k* = 100, as it was almost unaffected by the number of documents requested.

The third collection, **Influenza**, was the most surprising of the three. **PDL** with storing factor *β* set was between **Brute-L** and **Brute-D** in both time and space. We could not build **PDL** without the storing factor, as the document sets were too large for the Re-Pair compressor. The construction of **SURF** also failed with this dataset.

### Document counting

#### Indexes

We use two fast document listing algorithms as baseline document counting methods (see Sect. 7.2.1): **Brute-D** sorts the query range ${\textsf{DA}}[\ell ..r]$ to count the number of distinct document identifiers, and **PDL-RP** returns the length of the list of documents obtained. Both indexes use the RLCSA with suffix array sample period set to 32 on non-repetitive datasets, and to 128 on repetitive datasets.

We also consider a number of encodings of Sadakane’s document counting structure (see Sect. 5). The following ones encode the bitvector *H*′ directly in a number of ways:

- **Sada** uses a plain bitvector representation.
- **Sada-RR** uses a run-length encoded bitvector as supplied in the RLCSA implementation. It uses *δ*-codes to represent run lengths and packs them into blocks of 32 bytes of encoded data. Each block stores how many bits and 1s are there before it.
- **Sada-RS** uses a run-length encoded bitvector, represented with a sparse bitmap (Okanohara and Sadakane 2007) marking the beginnings of the 0-runs and another for the 1-runs.
- **Sada-RD** uses run-length encoding with *δ*-codes to represent the lengths. Each block in the bitvector contains the encoding of 128 1-bits, while three sparse bitmaps are used to mark the number of bits, 1-bits, and starting positions of block encodings.
- **Sada-Gr** uses a grammar-compressed bitvector (Navarro and Ordóñez 2014).

The following encodings use filters in addition to bitvector *H*′:

- **Sada-P-G** uses **Sada** for *H*′ and a gap-encoded bitvector for the filter bitvector *F*. The gap-encoded bitvector is also provided in the RLCSA implementation. It differs from the run-length encoded bitvector by only encoding runs of 0-bits.
- **Sada-P-RR** uses **Sada** for *H*′ and **Sada-RR** for *F*.
- **Sada-RR-G** uses **Sada-RR** for *H*′ and a gap-encoded bitvector for *F*.
- **Sada-RR-RR** uses **Sada-RR** for both *H*′ and *F*.
- **Sada-S** uses sparse bitmaps for both *H*′ and the sparse filter *F*_*S*_.
- **Sada-S-S** is **Sada-S** with an additional sparse bitmap for the 1-filter *F*_1_
- **Sada-RS-S** uses **Sada-RS** for *H*′ and a sparse bitmap for *F*_1_.
- **Sada-RD-S** uses **Sada-RD** for *H*′ and a sparse bitmap for *F*_1_.

Finally, **ILCP** implements the technique described in Sect. 3.4, using the same encoding as in **Sada-RS** to represent the bitvectors in the wavelet tree.

Our implementations of the above methods can be found online.^14^

#### Results

**Fig. 11:**
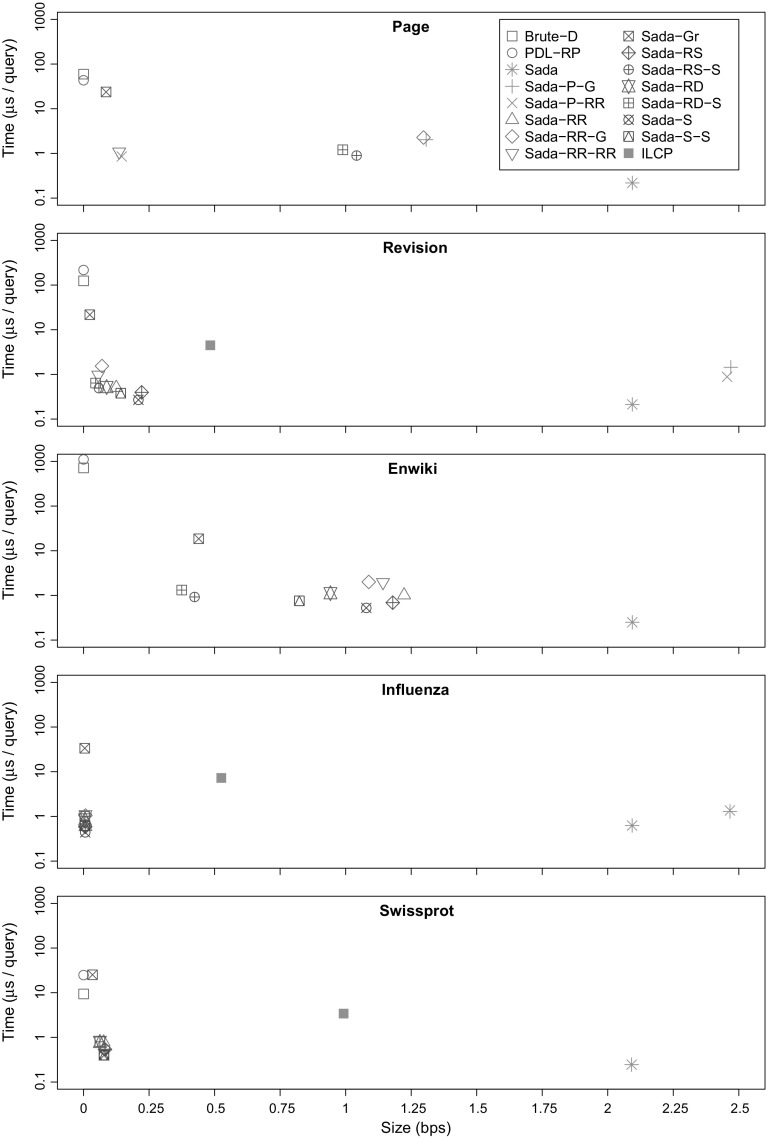
Document counting on different datasets. The size of the counting structure in bits per symbol (x) and the average query time in microseconds (y). The baseline document listing methods are presented as having size 0, as they take advantage of the existing functionalities in the index

Due to the use of 32-bit variables in some of the implementations, we could not build all structures for the large real collections. Hence we used the medium versions of **Page**, **Revision**, and **Enwiki**, the large version of **Influenza**, and the only version of **Swissprot** for the benchmarks. We started the queries from precomputed lexicographic ranges [ℓ..*r*] in order to emphasize the differences between the fastest variants. For the same reason, we also left out of the plots the size of the RLCSA and the possible document retrieval structures. Finally, as it was almost always the fastest method, we scaled the plots to leave out anything much larger than plain **Sada**. The results can be seen in Fig. 11. Table 5 in “Appendix 1” lists the results in further detail.

On **Page**, the filtered methods **Sada-P-RR** and **Sada-RR-RR** are clearly the best choices, being only slightly larger than the baselines and orders of magnitude faster. Plain **Sada** is much faster than those, but it takes much more space than all the other indexes. Only **Sada-Gr** compresses the structure better, but it is almost as slow as the baselines.

On **Revision**, there were many small encodings with similar performance. Among those, **Sada-RS-S** is the fastest. **Sada-S** is somewhat larger and faster. As on **Page**, plain **Sada** is even faster, but it takes much more space.

The situation changes on the non-repetitive **Enwiki**. Only **Sada-RD-S**, **Sada-RS-S**, and **Sada-Gr** can compress the bitvector clearly below 1 bit per symbol, and **Sada-Gr** is much slower than the other two. At around 1 bit per symbol, **Sada-S** is again the fastest option. Plain **Sada** requires twice as much space as **Sada-S**, but is also twice as fast.

**Influenza** and **Swissprot** contain, respectively, RNA and protein sequences, making each individual document quite random. Such collections are easy cases for Sadakane’s method, and many encodings compress the bitvector very well. In both cases, **Sada-S** was the fastest small encoding. On **Influenza**, the small encodings fit in CPU cache, making them often faster than plain **Sada**.

Different compression techniques succeed with different collections, for different reasons, which complicates a simple recommendation for a best option. Plain **Sada** is always fast, while **Sada-S** is usually smaller without sacrificing too much performance. When more space-efficient solutions are required, the right choice depends on the type of the collection. Our ILCP-based structure, **ILCP**, also outperforms **Sada** in space on most collections, but it is always significantly larger and slower than compressed variants of **Sada**.

### The multi-term ${\textsf{tf}}{\textsf{-}}{\textsf{idf}}$ index

We implement our multi-term index as follows. We use RLCSA as the ${\textsf{CSA}}$, **PDL-256+F** for single-term top-*k* retrieval, and **Sada-S** for document counting. We could have integrated the document counts into the **PDL** structure, but a separate counting structure makes the index more flexible. Additionally, encoding the number of redundant documents in each internal node of the suffix tree (**Sada**) often takes less space than encoding the total number of documents in each node of the sampled suffix tree (**PDL**). We use the basic ${\textsf{tf}}{\textsf{-}}{\textsf{idf}}$ scoring scheme.

We tested the resulting performance on the 1432 MB **Wiki** collection. RLCSA took 0.73 bps with sample period 128 (the sample period did not have a significant impact on query performance), **PDL-256+F** took 3.37 bps, and **Sada-S** took 0.13 bps, for a total of 4.23 bps (757 MB). Out of the total of 100,000 queries in the query set, there were matches for 31,417 conjunctive queries and 97,774 disjunctive queries.

The results can be seen in Table 3. When using a single query thread, the index can process 136–229 queries per second (around 4–7 ms per query), depending on the query type and the value of *k*. Disjunctive queries are faster than conjunctive queries, while larger values of *k* do not increase query times significantly. Note that our ranked disjunctive query algorithm preempts the processing of the lists of the patterns, whereas in the conjunctive ones we are forced to expand the full document lists for all the patterns; this is why the former are faster. The speedup from using 32 threads is around 18x.

**Table 3:** Ranked multi-term queries on the **Wiki** collection

Query	*k*	1 thread	8 threads	16 threads	32 threads
Ranked-AND	10	152	914	1699	2668
	100	136	862	1523	2401
Ranked-OR	10	229	1529	2734	4179
	100	163	1089	1905	2919

Query type, number of documents requested, and the average number of queries per second with 1, 8, 16, and 32 query threads

Since our multi-term index offers a functionality similar to basic inverted index queries, it seems sensible to compare it to an inverted index designed for natural language texts. For this purpose, we indexed the **Wiki** collection using **Terrier** (Macdonald et al. 2012) version 4.1 with the default settings. See Table 4 for a comparison between the two indexes.

Note that the similarity in the functionality is only superficial: our index can find *any text substring*, whereas the inverted index can only look for indexed *words and phrases*. Thus our index has an index point per symbol, whereas **Terrier** has an index point per word (in addition, inverted indexes usually discard words deemed uninteresting, like stopwords). Note that **PDL** also chooses frequent strings and builds their lists of documents, but since it has many more index points, its posting lists are 200 times longer than those of **Terrier**, and the number of lists is 300 times larger. Thanks to the compression of its lists, however, **PDL** uses only 8 times more space than **Terrier**. On the other hand, both indexes have similar query performance. When logging and output was set to minimum, **Terrier** could process 231 top-10 queries and 228 top-100 queries per second under the ${\textsf{tf}}{\textsf{-}}{\textsf{idf}}$ scoring model using a single query thread.

**Table 4:** Our index (**PDL**) and an inverted index (**Terrier**) on the **Wiki** collection

Index	Vocabulary	Posting lists	Collection	Size (MB)	Queries/s	
**PDL**	39.2M substrings	8840M documents	1500M symbols	757	229 (*k* = 10)	163 (*k* = 100)
**Terrier**	0.134M tokens	42.3M documents	133M tokens	90.1	231 (*k *= 10)	228 (*k* = 100)

The size of the vocabulary, the posting lists, and the collection in millions of elements, the size of the index in megabytes, and the number of Ranked-OR queries per second with *k* = 10 or 100 using a single thread

## Conclusions

We have investigated the space/time tradeoffs involved in indexing highly repetitive string collections, with the goal of performing information retrieval tasks on them. Particularly, we considered the problems of document listing, top-*k* retrieval, and document counting. We have developed new indexes that perform particularly well on those types of collections, and studied how other existing data structures perform in this scenario, and in which cases the indexes are actually better than brute-force approaches. As a result, we offered recommendations on which structures to use depending on the kind of repetitiveness involved and the desired space usage. As a proof of concept, we have shown how the tools we developed can be assembled to build an efficient index supporting ranked multi-term queries on repetitive string collections.

We do not aim to outperform inverted indexes on natural language text collecions, where they are unbeatable, but rather to offer similar capabilities on generic string collections, where inverted indexes cannot be applied. Our developments are at the level of algorithmic ideas and prototypes. In order to have our most promising structures scale up to real-world information systems, where inverted indexes are now the norm, various research problems must be faced:

1. Our construction algorithms scale up to a few gigabytes. This limits the collection sizes we can handle, even if they are repetitive and thus the final structures are much smaller. For example, our PDL structure first builds the classical suffix tree and then samples it. Using construction space proportional to that of the final structures in the case of repetitive scenarios, or building efficiently using the disk, is an important research problem.
2. When the datasets are sufficiently large, even the compressed structures will have to operate on disk. Inverted indexes are extremely disk-friendly, which makes them perform well on huge text collections. We have not yet studied this aspect of our structures, although PDL seems well-suited to this case: it traverses one or a few contiguous lists (which should be decompressed in main memory) or a contiguous area of the suffix array.
3. Our data structures are static, that is, they must be rebuilt from scratch when documents are inserted in the collection or deleted from it. Inverted indexes tolerate updates much better, though they are not fully dynamic either. Instead, since in many scenarios updates are not so frequent, popular solutions combine a large part of the collection that is indexed and a small recent part that is traversed sequentially. It is likely that our structures will also perform well under such a scheme, as long as we manage to rebuild the index periodically within controlled space and time.
4. We showed that our structures can handle multi-term queries under the simple ${\textsf{tf}}{\textsf{-}}{\textsf{idf}}$ scoring scheme. While this can be acceptable in some applications for generic string collections, information retrieval on natural language texts uses, nowadays, much more sophisticated formulas. Inverted indexes have been adapted to successfully support those formulas that are used for a first filtration step, such as BM25. Studying how to extend our indexes to handle these is another interesting research problem.
5. One point where our indexes could outperform inverted indexes is in phrase queries, where inverted indexes must perform costly list intersections. Our suffix-array based indexes, instead, need not do anything special. For a fair comparison, we should regard the text as a sequence of tokens (i.e., the terms that are indexed by the inverted index) and build our indexes on them. The resulting structure would then only answer term and phrase queries, just like an inverted index, but would be must faster at phrases.

## Appendix 1: Detailed results

Table 5 shows the precise numerical results displayed in Fig. 11, to allow for a finer-grained comparison.

**Table 5:** Document counting on different datasets

	**Page**	**Revision**	**Enwiki**	**Influenza**	**Swissprot**
**Brute-D**	59.419 µs	**124.286** µs	**714.481** µs	**4557.310** µs	**9.392** µs
	0.000 b	**0.000** b	**0.000** b	**0.000** b	**0.000** b
**PDL-RP**	**43.356** µs	217.804 µs	1107.470 µs	6221.610 µs	24.848 µs
	**0.000** b	0.000 b	0.000 b	0.000 b	0.000 b
**Sada**	**0.218** µs	**0.213** µs	**0.250** µs	0.624 µs	**0.246** µs
	**2.094** b	**2.094** b	**2.094** b	2.093 b	**2.091** b
**Sada-P-G**	2.030 µs	1.442 µs	1.608 µs	1.291 µs	–
	1.307 b	2.469 b	2.694 b	2.466 b	–
**Sada-P-RR**	**0.852** µs	0.882 µs	1.572 µs	1.356 µs	–
	**0.146** b	2.455 b	2.748 b	2.466 b	–
**Sada-RR**	1.105 µs	0.506 µs	1.013 µs	0.581 µs	**0.779** µs
	5.885 b	0.125 b	1.223 b	0.007 b	**0.076** b
**Sada-RR-G**	2.268 µs	1.535 µs	2.001 µs	1.046 µs	–
	1.297 b	0.070 b	1.088 b	0.007 b	–
**Sada-RR-RR**	**1.088** µs	0.974 µs	1.960 µs	1.108 µs	–
	**0.136** b	0.056 b	1.142 b	0.007 b	–
**Sada-Gr**	23.750 µs	**21.643** µs	18.542 µs	33.502 µs	**25.236** µs
	0.086 b	**0.024** b	0.439 b	0.005 b	**0.034** b
**Sada-RS**	0.742 µs	0.396 µs	0.688 µs	0.584 µs	0.538 µs
	5.991 b	0.222 b	1.180 b	0.006 b	0.082 b
**Sada-RS-S**	0.897 µs	**0.492** µs	**0.923** µs	**0.767** µs	0.545 µs
	1.042 b	**0.059** b	**0.424** b	**0.005** b	0.082 b
**Sada-RD**	1.019 µs	0.521 µs	1.119 µs	0.856 µs	**0.792** µs
	3.717 b	0.088 b	0.942 b	0.006 b	**0.062** b
**Sada-RD-S**	1.205 µs	**0.641** µs	**1.316** µs	1.005 µs	0.799 µs
	0.989 b	**0.046** b	**0.374** b	0.005 b	0.062 b
**Sada-S**	0.604 µs	**0.269** µs	**0.525** µs	**0.439** µs	**0.396** µs
	5.729 b	**0.209** b	**1.079** b	**0.006** b	**0.078** b
**Sada-S-S**	0.735 µs	**0.380** µs	**0.755** µs	0.624 µs	0.399 µs
	3.432 b	**0.142** b	**0.823** b	0.006 b	0.078 b
**ILCP**	4.399 µs	4.482 µs	6.033 µs	7.252 µs	3.414 µs
	18.454 b	0.484 b	4.575 b	0.525 b	0.992 b

The average query time in microseconds and the size of the counting structure in bits per symbol. Results on the Pareto frontier have been highlighted. The baseline document listing methods **Brute-D** and **PDL-RP** are presented as having size 0, as they take advantage of the existing functionalities in the index. We did not build **Sada-P-G**, **Sada-P-RR**, **Sada-RR-G**, and **Sada-RR-RR** for **Swissprot**, because the filter was empty and the remaining structure was equivalent to **Sada** or **Sada-RR**

## Appendix 2: Index construction

Our construction algorithms prioritize flexibility over performance. For example, the construction of the ${\textsf{tf}}{\textsf{-}}{\textsf{idf}}$ index (Sect. 6) proceeds as follows:

1. Build RLCSA for the collection.
2. Extract the LCP array and the document array from the RLCSA, traverse the suffix tree by using the LCP array, and build **PDL** with uncompressed document sets.
3. Compress the document sets using a Re-Pair compressor.
4. Build the **Sada-S** structure using a similar algorithm as for **PDL** construction.

See Table 6 for the time and space requirements of building the index for the **Wiki** collection.

**Table 6:** Building the ${\textsf{tf}}{\textsf{-}}{\textsf{idf}}$ index for the **Wiki** collection

	RLCSA	**PDL**	Re-Pair	**Sada-S**	Total
Time (min)	10.5	39.2	123	74.7	248
Memory (GB)	19.6	111	202	92.8	202

Construction time in minutes and peak memory usage in gigabytes for RLCSA construction, **PDL** construction, compressing the document sets using Re-Pair, **Sada-S** construction, and the entire construction

Scaling the index up for larger collections requires faster and more space-efficient construction algorithms for its components. There are some obvious improvements:

- RLCSA construction can be done in less memory by building the index in multiple parts and merging the partial indexes (Sirén 2009). With 100 parts, the indexing of a repetitive collection proceeds at about 1 MB/s using 2–3 bits per symbol (Sirén 2012). Newer suffix array construction algorithms achieve even better time/space trade-offs (Kärkkäinen et al. 2015).
- We can use a compressed suffix tree for **PDL** construction. The SDSL library (Gog et al. 2014) provides fast scalable implementations that require around 2 bytes per symbol.
- We can write the uncompressed document sets to disk as soon as the traversal returns to the parent node.
- We can build the *H* array for **Sada-S** by keeping track of the lowest common ancestor of the previous occurrence of each document identifier and the current node. If node *v* is the lowest common ancestor of consecutive occurrences of a document identifier, we increment the corresponding cell of the *H* array. Storing the array requires about a byte per symbol.

The main bottleneck in the construction is Re-Pair compression. Our compressor requires 24 bytes of memory for each integer in the document sets, and the number of integers (8.9 billion) is several times larger than the number of symbols in the collection (1.5 billion). It might be possible to improve compression performance by using a specialized compressor. If interval ${\textsf{DA}}[\ell ..r]$ corresponds to suffix tree node *u* and the collection is repetitive, it is likely that the interval ${\textsf{DA}}[\ell '..r']$ corresponding to the node reached by taking the suffix link from *u* is very similar to ${\textsf{DA}}[\ell ..r]$.

## Footnotes

1
www.wikipedia.org.

2 From the Internet Archive, www.archive.org/web/web.php.

3 Such as p7zip, http://p7zip.sourceforge.net.

4 This is achieved by using a constant-time ${{\textsf{rank}}}$/${{\textsf{select}}}$ solution (Clark 1996) to represent their internal bitvector *H*.

5 This model states that the statistical dependence of a symbol from previous ones tends to zero as the distance towards them tends to infinity. The A2 model includes, in particular, the Bernoulli model (where each symbol is generated independently of the context), stationary Markov chains (where the probability of each symbol depends on the previous one), and *k*th order models (where each symbol depends on the *k* previous ones, for a fixed *k*).

6 This is a very strong kind of convergence. A sequence *X*_*n*_ tends to a value *β* almost surely if, for every $\epsilon > 0$, the probability that $|X_N/\beta -1 | > \epsilon$ for some *N* > *n* tends to zero as *n* tends to infinity, $\lim _{n\rightarrow \infty }\sup _{N>n} \mathrm {Pr}(|X_N/\beta -1 |>\epsilon )=0$.

7 See http://jltsiren.kapsi.fi/rlcsa for the datasets and full results.

8
http://pizzachili.dcc.uchile.cl.

9
http://trec.nist.gov/data/terabyte06.html.

10
http://www.dcc.uchile.cl/gnavarro/software.

11
http://jltsiren.kapsi.fi/rlcsa.

12
http://jltsiren.kapsi.fi/rlcsa.

13
http://github.com/simongog/surf/tree/single_term.

14
http://jltsiren.kapsi.fi/rlcsa and http://github.com/ahartik/succinct.
